# Systematic review of reviews on interventions to engage men and boys as clients, partners and agents of change for improved sexual and reproductive health and rights

**DOI:** 10.1136/bmjopen-2024-083950

**Published:** 2025-01-20

**Authors:** Ann Gottert, Julie Pulerwitz, Renay Weiner, Chantalle Okondo, Jesse Werner, Sarah Magni, Sanyukta Mathur

**Affiliations:** 1 Population Council, Washington, District of Columbia, USA; 2 Genesis Analytics Pty Ltd, Johannesburg, Gauteng, South Africa; 3 University of the Witwatersrand Johannesburg, Johannesburg, Gauteng, South Africa; 4 Research and Training for Health and Development, Johannesburg, Gauteng, South Africa; 5 Population Council Kenya, Nairobi, Kenya

**Keywords:** Systematic Review, Health Education, Primary Prevention, Psychosocial Intervention, REPRODUCTIVE MEDICINE

## Abstract

**Abstract:**

**Objectives:**

Evidence regarding interventions to engage men and boys to improve sexual and reproductive health and rights (SRHR) has grown rapidly across subtopics such as HIV, family planning and gender-based violence (GBV). We conducted a review of the effectiveness of interventions to engage men and boys across SRHR domains, lessons learnt about successful programming, and about harms/unintended consequences, in low- and middle-income countries (LMIC).

**Design:**

Systematic review of reviews following Cochrane guidelines.

**Data sources:**

PubMed, Cumulative Index to Nursing and Allied Health Literature, Web of Science and Cochrane were searched (18 October–9 November 2022; 9 September 2024).

**Eligibility criteria:**

Eligible reviews were published since 2015, covered WHO-defined SRHR domain(s) and focused mostly on LMIC.

**Data extraction and synthesis:**

Data extraction by multiple reviewers focused on intervention effectiveness, implementation best practices, unintended consequences, and quality/risk of bias.

**Results:**

Thirty-five systematic reviews were included, comprising ~960 primary studies. Reviews focused on HIV prevention/care, reproductive health, maternal and newborn health, and GBV. Reviews consistently concluded that men were successfully engaged, yielding benefits to both women and men’s SRHR outcomes; no adverse intervention impacts on prevalence of SRHR outcomes were reported. We summarised the interventions most consistently found to be effective across reviews, in a programmer-friendly visual mapped onto a framework of men as clients, partners and agents of change. Person-centred, gender-transformative, multilevel approaches were most effective. Remaining evidence gaps include engaging men as contraceptive *users*, sexually transmitted infections other than HIV, preventing unsafe abortion and SGBV as experienced by men and boys.

**Conclusions:**

There is substantial evidence supporting a range of successful interventions to engage men and boys to improve SRHR, with markedly similar principles and approaches emerging across SRHR domains. It is time to scale up and integrate these strategies, monitoring for any potential harms and tailoring as needed to socio-cultural contexts and for specific vulnerable subpopulations.

**STRENGTHS AND LIMITATIONS OF THIS STUDY**

- This systematic review of 35 reviews brings together the evidence regarding interventions to engage men and boys to improve sexual and reproductive health and rights (SRHR) in low and middle-income countries.
- The authors map out which male engagement interventions and implementation strategies have consistently been shown to be effective across SRHR domains.
- The methodology used was robust and included a comprehensive search strategy organised based on an established framework for male engagement, as well as data screening, extraction and synthesis by multiple experienced reviewers.
- This systematic review of reviews was necessarily reliant on the objectives and scope of the included reviews.
- The search was limited to reviews available in English in the peer-reviewed literature, which may have resulted in exclusion of certain otherwise relevant reviews.

## Introduction

 Building on discussions that started at the global population and development meetings in Cairo (1994) and Beijing (1995), there is now widespread recognition that men and boys’ engagement in sexual and reproductive health and rights (SRHR) is crucial for achieving equitable outcomes for all. This includes outcomes for men and boys themselves, who also face SRHR-related health concerns, such as high HIV-related morbidity and mortality and sexual violence.^1^,^5^ It also includes outcomes for their female partners, especially adolescent girls and young women who are often additionally vulnerable due to inequitable gendered relationship power dynamics, as well as outcomes for their children. Engaging men and boys, also referred to as ‘male engagement’ and sometimes ‘male involvement’, entails the active participation, involvement and inclusion of men and boys in a wide range of SRHR practices, programmes and policies, such as accompanying female partners to antenatal visits, using pre-exposure prophylaxis (PrEP) to prevent HIV acquisition for themselves and their partners, and engaging in critical reflection and advocacy around gender-based violence (GBV). Effectively engaging men and boys in SRHR seeks to break down barriers that may limit their involvement and facilitates their roles as supportive partners, advocates, and allies in promoting SRHR of all individuals.

Sexual and reproductive health can be defined as ‘…a state of physical, emotional, mental and social well-being in relation to all aspects of sexuality and reproduction, not merely the absence of disease, dysfunction or infirmity… All individuals have a right to make decisions governing their bodies and to access services that support that right. Achievement of sexual and reproductive health relies on the realisation of sexual and reproductive rights’.^6^ The importance of engaging men and boys has been increasingly embraced within multiple domains under the SRHR umbrella, for instance, related to HIV prevention, contraception/family planning, maternal and newborn health, and preventing GBV. In parallel, the evidence base for effective interventions to engage men and boys in these areas has greatly expanded. A 2010 review of 58 evaluation studies addressing male engagement in a range of SRHR issues reported promising results but found that interventions tended to be short and few programmes went beyond the pilot stage.^7^ Since then, dozens of systematic reviews on male engagement to improve SRHR have been published. Still, the literature, including systematic reviews, remains largely siloed within SRHR domains, obscuring potentially informative commonalities. There also remains substantial uncertainty regarding which male engagement intervention strategies are effective as well as best practices for implementing these strategies—particularly among institutions charged with promoting SRHR in a given country—and an overarching framework for these strategies is needed. Questions also persist about potential unintended consequences on women’s autonomy and SRHR.

We conducted a systematic review of reviews to elucidate lessons learnt about effectiveness of different types of programming to engage men and boys (ages 10 years or older), in different geographic regions, and by SRHR domains/outcomes, while also assessing any reports of harms or unintended consequences. Systematic reviews of reviews aim to summarise evidence across multiple systematic reviews, as opposed to summarising evidence across multiple individual primary studies/papers.^8^ We did not include reviews focused on sexual and/or gender minority men’s engagement in SRHR, since there is another sizeable literature on this topic, and the types of programming, and effectiveness, may differ for these populations. This research was part of a project focused on translating HIV prevention evidence into action in sub-Saharan Africa (currently called insight2implementation), which is part of the South-to-South HIV Prevention Learning Network (SSLN). SSLN stakeholders from multiple countries in Eastern and Southern Africa identified a need for evidence synthesis on strategies to engage men and boys as a priority. To organise our review, we applied an existing framework on male engagement that delineates programming for: (1) men and boys as clients, to meet their own healthcare and prevention needs; (2) men as partners, to engage them as equitable and supportive intimate partners; and (3) men as agents of change, to create enabling environments that promote gender equity and health.^9^

## Methods

For our review, we followed the Cochrane guidelines for how to conduct overviews (ie, systematic reviews of reviews).^8^ Ethical approval was not required for this review as it did not entail research on human subjects as patients or informants. The review of reviews is registered with Research Registry (unique identification number (UIN): reviewregistry1884).

### Search and eligibility criteria

We conducted two searches (18 October to 9 November 2022 and 9 September 2024 (to capture more recent reviews)) using the following databases: PubMed, Cochrane Database of Systematic Reviews, Cumulative Index to Nursing and Allied Health Literature and Web of Science. A summary of the database searches, including search terms used, is included in online supplemental file 1. We conducted supplementary searching to identify any further reviews which had not been found in the database searches, including hand-searches of reference lists of included articles.

For inclusion, the review had to be systematic in nature, which we defined as meeting all of the following criteria:

1. The a priori specification of a research question (ie, at least one research question was clearly stated and identified as motivating the review subsequently undertaken, in the paper’s introduction or methods section).
2. Clarity on the scope of the review and which studies are eligible for inclusion.
3. Making every effort to find all relevant research and to ensure that issues of bias in included studies are accounted for.
4. Analysing the included studies in order to draw conclusions based on all the identified research in an impartial and objective way.

Additional inclusion criteria were as follows:

- Publication between 2015 and 2023 in the peer-reviewed literature, in English.
- At least half of the primary studies from low- and middle-income countries per World Bank definition^10^ (or conduct separate analysis of evidence from low- and middle-income countries).
- Focused on interventions that explicitly sought to engage men and/or boys ages 10 years and older.
- Covered at least one of seven SRHR domains. These domains included: *helping people realise their desired family size; ensuring the health of pregnant women/girls and their newborn infants; preventing unsafe abortion; promoting sexual health and well-being; promoting SRHR in disease outbreaks; promoting healthy adolescence for a healthy future; and preventing and responding to gender-based violence and harmful practices*. Domains were defined based on outcomes of interest per the WHO Reproductive Health Strategy, as employed by Ruane-McAteer *et al* in their WHO-commissioned ‘evidence and gap map’ of interventions engaging men and boys in SRHR.^11 12^

Reviews could include studies with quantitative or qualitative methods or both. An exclusion criterion was if the review primarily focused on sexual and/or gender minority populations, for the reasons described above.

### Title/abstract and full-text review

Search results were entered into Covidence, a web-based collaboration platform to manage systematic reviews.^13^ Two reviewers double-screened titles/abstracts until consensus was reached; the remaining titles/abstracts were then single-screened. Full-text reviews were then conducted by at least two reviewers, with discussion in case of disagreements until consensus was reached. All reviewers have experience with SRHR programming and research, as well as synthesis of SRHR evidence, in sub-Saharan Africa, and globally (for several reviewers). AG and JP each have 20+ years of experience developing, implementing and evaluating programmes to engage men and boys in SRHR worldwide have developed frameworks (including the men as clients/partners/agents of change framework employed in this review), and have published extensively regarding male engagement in SRHR.^49 14^,^25^

### Data extraction and analysis

Data extraction was conducted by five reviewers using the Extraction 2.0 tool in Covidence. The data extraction template was developed by AG and JP and then reviewed and refined with the co-authors to ensure clarity and consistency. The extraction template included 26 fields, comprising both closed-ended fields and open-ended/text fields. Information from the close-ended fields (listed in the first column of table 2) was summarised across reviews using counts and summary statistics (eg, frequencies, means). Open-ended/text fields included the review’s conclusions regarding: intervention effectiveness; implementation best practices; contextual variation in effectiveness/implementation; risks and unintended consequences; study quality/risk of bias; and future focus for interventions and research. Three authors conducted the first data extraction and quality appraisal; two other authors (AG, JP) independently read included articles and reviewed/refined data extraction and quality appraisal entries. These two authors then independently reviewed the open-ended/text-based data extraction outputs (with reviews grouped into men as clients/partners/agents of change) to identify emerging themes within each of these groupings. The full study team then met together to reach consensus on themes for each of the open-ended/text fields listed above (eg, intervention effectiveness, implementation best practices, etc).

Finally, we sought to develop a summative conceptual framework that identifies the types of male engagement interventions and strategies most consistently found to be effective across the reviews, mapping onto the men as clients/partners/agents of change categories, as well as different levels of the social ecological framework.^26^ This conceptual framework is intended to be a future reference for programmers, researchers, policymakers, and other stakeholders who share the goal of improving SRHR for all.

### Assessing review quality and risk of bias

Along with conducting data extraction (which included summarising reviews’ conclusions about of quality/risk of bias for the primary studies they included), two reviewers per article also rated the *quality/risk of bias of each systematic review* according to 11 criteria (plus one additional criterion for meta-analyses), following A Measurement Tool to Assess Systematic Reviews, version 2 (AMSTAR 2) tool.^27^ We also assessed the degree of overlap in primary papers included across the reviews, to identify any papers that may be over-represented across reviews (hence potentially biasing findings toward those of those papers).

### Patient and public involvement

SSLN stakeholders from multiple countries in Eastern and Southern Africa identified a need for evidence synthesis on strategies to engage men and boys as a priority. That request was the impetus for this review of reviews. Emerging themes from the analysis of data extraction tables were shared with country champions and other stakeholders in the SSLN, including community members, sexual and reproductive health (SRH) service providers and government representatives, during a global webinar on 1 August 2023 at which reflections and feedback were gathered. Feedback from local stakeholders included a request for a simplified presentation of the findings and guidance on how to integrate these findings into policy and programmes. The study team then integrated this feedback into the final findings presented in this manuscript.

### Role of the funding source

The funder of the study had no role in study design, data collection, data analysis, data interpretation or writing of the paper.

## Results

**Figure 1:**
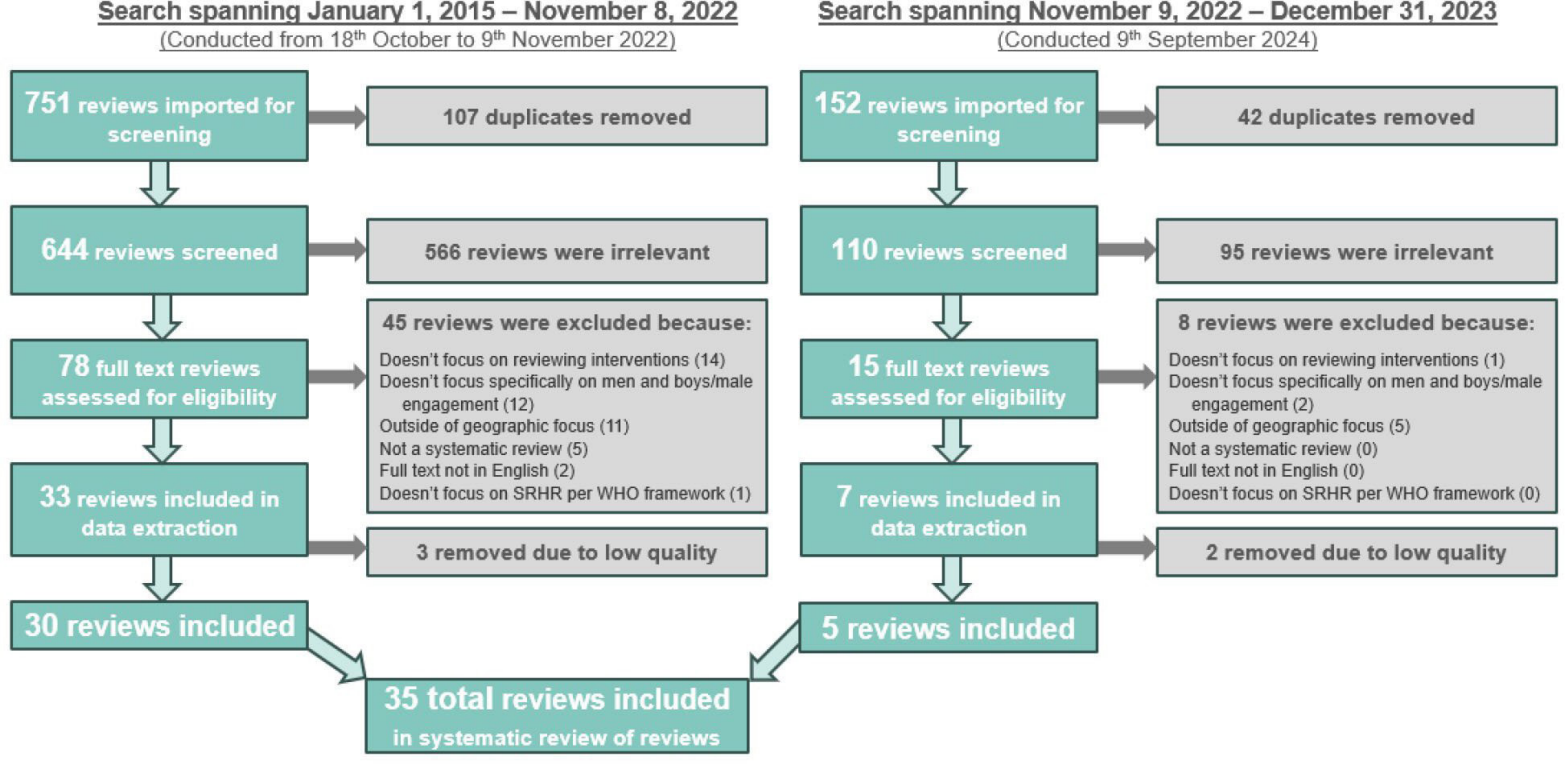
Preferred Reporting Items for Systematic Reviews and Meta-Analyses (PRISMA) diagram. SRHR, sexual and reproductive health and rights.

Figure 1 shows a Preferred Reporting Items for Systematic Reviews and Meta-Analyses (PRISMA) diagram. In total (across the first and second searches), 903 reviews were imported for screening, of which 149 duplicates were removed. Of the remaining 754 review titles and abstracts screened, 661 did not meet inclusion criteria. Of the 93 full-text reviews screened, 53 were excluded. Five reviews initially included in the set of final reviews, were subsequently rated as having low quality during the quality assessment (met five or fewer of the 11 criteria) and hence were dropped from the final analysis. A list of reviews excluded at or after the full-text review stage, and main reason for exclusion, is included in online supplemental table 1. This yielded a final total of 35 systematic reviews.

### Characteristics of included reviews

Table 1 lists the 35 included systematic reviews, along with the number, methodology, geographic coverage and timespan of primary studies included.

**Table 1:** List of included reviews

Author(s), yearand journal	Title	# Primary studies	Methodology of included studies	Geographic coverage	Timespan
ENGAGING MEN AS CLIENTS					
Kaufman *et al*, 2016 *PLoS ONE*^29^	Adolescent sexual and reproductive health services and implications for the provision of voluntary medical male circumcision: results of a systematic literature review	79	Quantitative: any evaluation design; qualitative	Africa	1990–2014
Shattuck *et al*, 2016 *Global Health: Science & Practice*^30^	A review of 10 years of vasectomy programming and research in low-resource settings	75	Quantitative: any evaluation design; qualitative	Africa; Asia; LAC	2005–2015
Hardee *et al*, 2017 *Reproductive Health*^31^	Are men well served by family planning programs?	47	Quantitative: any evaluation design; qualitative	Africa; Asia; Europe; LAC	2010–2015
Ensor *et al*, 2019 *J. International AIDS Society*^32^	The effectiveness of demand creation interventions for voluntary male medical circumcision for HIV prevention in sub-Saharan Africa: a mixed methods systematic review	18	Quantitative: any evaluation design; qualitative	Africa	Up to 2018
Hlongwa *et al*, 2019 *BMC Infectious Diseases*^33^	Mapping evidence of intervention strategies to improving men’s uptake to HIV testing services in sub-Saharan Africa: a systematic scoping review	24	Quantitative: any evaluation design; qualitative	Africa	1990–2018
Hlongwa *et al*, 2020 *BMC Public Health*^34^	Men’s perspectives on HIV self-testing in sub-Saharan Africa: a systematic review and meta-synthesis	16	Qualitative	Africa	2005–2019
Atkins *et al*, 2020 *PLoS ONE*^35^	Service delivery interventions to increase uptake of voluntary medical male circumcision for HIV prevention: a systematic review	29	Quantitative: any evaluation design; qualitative	Africa	1990–2018
Kusemererwa *et al*, 2021 *PLoS ONE*^36^	Strategies for retention of heterosexual men in HIV care in sub-Saharan Africa: a systematic review	14	Quantitative: any evaluation design	Africa	2005–2020
Hamilton *et al*, 2021 *Frontiers in Public Health*^37^	HIV self-testing uptake and intervention strategies among men in sub-Saharan Africa: a systematic review	63	Quantitative: any evaluation design; qualitative	Africa	2010–2020
Hlongwa *et al*, 2022 *AIDS and Behavior*^38^	Linkage to HIV care following HIV self-testing among men: systematic review of quantitative and qualitative studies from six countries in sub-Saharan Africa	15	Quantitative: any evaluation design; qualitative	Africa	2005–2022
Kanyemba *et al*, 2023 *AIDS and Behavior*^39^	HIV focused sexual risk reduction interventions targeting adolescent boys and young men in sub-Saharan Africa: a scoping review	29	Quantitative: any evaluation design; qualitative	Africa	2000–2020
Ramraj *et al*, 2023 *BMJ Open*^40^	Service delivery models that promote linkages to PrEP for adolescent girls and young women and men in sub-Saharan Africa: a scoping review	37	Quantitative: any evaluation design; qualitative	Africa	Up to 2021
Dovel *et al*, 2023 *medRxiv*^41^	Interventions to improve men’s engagement in HIV and other key services in sub-Saharan Africa: a scoping review	71	Quantitative: any evaluation design	Africa	2009–2022
ENGAGING MEN AS PARTNERS					
Ambia *et al*, 2016 *J. International AIDS Society*^44^	A systematic review of interventions to improve prevention of mother-to-child HIV transmission service delivery and promote retention	34	Quantitative: experimental only	Africa; NA	Up to 2015
Takah *et al,* 2017 *BMJ Open*^45^	The impact of approaches in improving male partner involvement in the prevention of mother-to-child transmission of HIV on the uptake of maternal antiretroviral therapy among HIV-seropositive pregnant women in sub-Saharan Africa: a systematic review and meta-analysis	17	Quantitative: any evaluation design	Africa	Up to 2016
Takah *et al*, 2018 *PLoS ONE*^46^	The impact of approaches in improving male partner involvement in the prevention of mother-to-child transmission of HIV on the uptake of safe infant feeding practices by HIV positive women in sub-Saharan Africa. A systematic review and meta-analysis.	6	Quantitative: any evaluation design	Africa	Up to 2018
Mashaphu *et al*, 2018 *South African J. of Psychiatry*^47^	Psychosocial and behavioural interventions towards HIV risk reduction for serodiscordant couples in Africa: a systematic review	8	Quantitative: any evaluation design; qualitative	Africa	1990–2015
Tokhi *et al,* 2018 *PLoS ONE*^48^	Involving men to improve maternal and newborn health: a systematic review of the effectiveness of interventions	13	Quantitative: any evaluation design	Africa; Asia	2000–2012
Triulzi *et al*, 2019 *BMC Health Services Research*^49^	The effectiveness of interventions to involve men living with HIV positive pregnant women in low-income countries: a systematic review of the literature	12	Quantitative: any evaluation design	Africa	2008–2018
Takah *et al,* 2019 *Maternal and Child Health Journal*^50^	Male partner involvement in the utilization of hospital delivery services by pregnant women living with HIV in sub-Saharan Africa: a systematic review and meta-analysis	8	Quantitative: any evaluation design	Africa	2015–2018
Nkwonta *et al,* 2019 *Internat. Persp. Sexual and Rep. Health*^43^	Male participation in reproductive health interventions in sub-Saharan Africa: a scoping review	18	Quantitative: any evaluation design; qualitative	Africa	2007–2018
Hailemariam *et al*, 2020 *AIDS Care*^51^	Uptake of couples HIV testing and counselling among heterosexual couples in Sub-Saharan Africa: a systematic review and meta-analysis	14	Quantitative: any evaluation design	Africa	2000–2017
Forbes *et al,* 2021 *BMC Pregnancy and Childbirth*^52^	Male partner involvement in birth preparedness, complication readiness and obstetric emergencies in Sub-Saharan Africa: a scoping review	35	Quantitative: any evaluation design; qualitative	Africa	2006–2019
Hampanda *et al,* 2022 *AIDS and Behavior*^53^	A systematic review of behavioral couples-based interventions targeting prevention of mother-to-child transmission in low and middle-income countries	5	Quantitative: experimental only	Africa	Up to 2019
Aventin *et al,* 2023 *Campbell Systematic Reviews*^42^	Involving men and boys in family planning: a systematic review of the effective components and characteristics of complex interventions in low- and middle-income countries	127	Quantitative: any evaluation design; qualitative	Africa; Asia; LAC	Up to 2020
ENGAGING MEN AS AGENTS OF CHANGE / to create enabling environments for SRHR					
Bourey *et al,* 2015 *BMC Public Health*^54^	Systematic review of structural interventions for intimate partner violence in low- and middle-income countries: organizing evidence for prevention	16	Quantitative: any evaluation design	Africa; Asia; LAC	2000–2015
Ellsberg *et al*, 2015 *The Lancet*^55^	Prevention of violence against women and girls: what does the evidence say?	18 from LMIC	Quantitative: experimental only	Africa; Asia; Europe; LAC; NA; Oceania	Up to 2015
Ruane-McAteer *et al,* 2020 *BMJ Global Health*^56^	Gender-transformative programming with men and boys to improve sexual and reproductive health and rights: a systematic review of intervention studies	68	Quantitative: experimental only	Africa; Asia; Europe; LAC; NA	2007–2018
Levy *et al,* 2020 *Lancet Global Health*^57^	Characteristics of successful programmes targeting gender inequality and restrictive gender norms for the health and wellbeing of children, adolescents, and young adults: a systematic review	61	Quantitative: any evaluation design; qualitative	Africa; Asia; Europe; LAC; NA	2000–2018
Pérez-Martinez *et al*, 2021 *Trauma, Violence, & Abuse*^58^	Positive masculinities and gender-based violence educational interventions among young people: a systematic review	15	Quantitative: any evaluation design; qualitative	Africa; Asia; NA	2008–2019
DeHond *et al,* 2022 *Trauma, Violence and Abuse*^59^	Prevention of perpetration of intimate partner violence by men and boys in low and middle income countries: a scoping review of primary prevention interventions	16	Quantitative: any evaluation design	Africa; Asia; LAC	2001–2020
Sabri *et al,* 2022 *Trauma, Violence and Abuse*^60^	Gender-based violence interventions in low- and middle-income countries: a systematic review of interventions at structural, community, interpersonal, individual, and multiple levels	60	Quantitative: any evaluation design	Africa; Asia; LAC	2004–2021
Kirkwood *et al,* 2022 *J. Medical Internet Research*^61^	The role of mHealth interventions in changing gender relations: systematic review of qualitative findings	14	Qualitative	Africa; Asia	2013–2020
Keith *et al*, 2022 *Trauma, Violence, & Abuse*^62^	A systematic review of interventions to reduce gender-based violence among women and girls in sub-Saharan Africa	53	Quantitative: any evaluation design	Africa	2000–2021
Leight *et al,* 2023 *Journal of Global Health*^63^	Effectiveness of community mobilisation and group-based interventions for preventing intimate partner violence against women in low- and middle-income countries: a systematic review and meta-analysis	30	Quantitative: experimental only	Africa; Asia; LAC	Up to 2021

GBVgender-based violenceIPV, intimate partner violence; LAC, Latin America & Caribbean; LMIC, low and middle-income countries; NA, Northern AmericaPMTCT, prevention of mother-to-child transmission (of HIV)PrEPpre-exposure prophylaxisSRHRsexual and reproductive health and rightsVAWGviolence against women and girlsVMMCvoluntary medical male circumcision

Table 2 summarises characteristics of the reviews. There was a gradual increase in the number of systematic reviews published each year. In terms of geographic coverage, which was based on six United Nations regions, all (100%) of the reviews included Africa, 37% included Asia, 34% Latin America and the Caribbean, 11% Europe, 11% North America and 3% Oceania. Over half of reviews (n=20) were described as systematic reviews; six were systematic reviews and meta-analysis; one was a systematic review and qualitative meta-synthesis; six were scoping reviews; and two were other reviews (type unspecified).

**Table 2:** Characteristics of included reviews

	N out of 35	%
Year published		
2015	2	6%
2016	3	9%
2017	2	6%
2018	3	9%
2019	5	14%
2020	5	14%
2021	4	11%
2022	6	17%
2023	5	14%
Geographic regions*		
Africa	35	100%
Asia	13	37%
Europe	4	11%
Latin America and the Caribbean	12	34%
North America	4	11%
Oceania	1	3%
Type of review		
Systematic review	20	57%
Systematic review and meta-analysis	6	17%
Systematic review and qualitative meta-synthesis	1	3%
Scoping review	6	17%
Review (type unspecified)	2	6%
SHRH domain†		
Helping people realise their desired family size (contraception and family planning; prevention and treatment of infertility)	7	20%
Ensuring the health of pregnant women/girls and their newborn infants (maternal and infant mortality; preventing complications in pregnancy, childbirth and postnatal period))	14	40%
Preventing unsafe abortion	1	3%
Promoting sexual health and well-being (prevention of reproductive tract and sexually transmitted infections; HIV/AIDS; and promotion of sexual well-being and function)‡	23	66%
Promoting SRHR in disease outbreaks (including prevention of sexual transmission, eg, of Zika or Ebola viruses)	3	9%
Promoting healthy adolescence for a healthy future (covering all SRHR outcomes, with a specific focus on adolescents)	5	14%
Preventing and responding to gender-based violence and harmful practices (intimate partner violence, domestic violence and sexual coercion/violence; and harmful practices (ie, female genital mutilation; child, early and forced marriage))	11	31%
*Covered at least two SRHR domains*	*16*	*46%*
Number of primary studies included - mean (range, IQR)	33 (5–127; 15, 50)	
Methodology of included studies		
Quantitative only (of these, number limited to experimental designs)	17 (5/17)	49%
Quantitative and qualitative (mixed methods)	16	46%
Qualitative only	2	6%
Main focal population(s) for interventions		
Both adults and youth§	17	49%
Adults	11	31%
Youth	3	9%
Men and young men	4	11%
Extent to which studies collected data with men		
All/most	16	46%
Some	13	37%
Few/none	4	11%
Types of interventions		
Community education	27	77%
Health facility education	19	54%
Health facility service delivery approaches	20	57%
Community service delivery approaches	21	60%
Couples counselling	19	54%
Reaching men via their partners	17	49%
Mass media social mobilisation campaigns	15	43%
Gender transformative workshops	12	34%
School-based programmes	13	37%
Health facility strengthening/provider training	9	26%
Financial incentives	11	31%
Structural interventions	7	20%
mHealth interventions	8	23%
Workplace programmes	2	6%
Other	121	34%
Funding reported		
National Institutes of Health	5	14%
Bill & Melinda Gates Foundation	3	9%
USAID/PEPFAR	5	14%
WHO	4	11%
DFID/FCDO	3	9%
Other	14	40%
None reported	12	34%

* Per United Nations regions.

† WHO SRHR domains.

‡ Excluding conditions not acquired sexually, for example, testicular and prostate cancers.

§ By ‘youth’/‘”young men’ is meant ages 10–24.

DFIDDepartment for International DevelopmentFCDOForeign, Commonwealth & Development OfficePEPFARU.S. President's Emergency Plan for AIDS ReliefSRHRsexual and reproductive health and rightsUSAIDUnited States Agency for International Development

Over half (54%) of reviews covered one SRHR domain, while 46% covered two or more. Most common were promoting sexual health and well-being (including HIV prevention; 66%), followed by ensuring the health of pregnant women/girls and their newborn infants (40%), promoting and responding to violence against women/girls (31%), and helping people realise their desired family size (20%).

On average, reviews included 33 primary studies (range 5–127; IQR 15, 50), with 1080 primary studies in total, of which 966 were non-overlapping. About half (49%) of reviews only included quantitative studies (with four of these limited to experimental designs only), 46% included both quantitative and qualitative studies, and two reviews only included qualitative studies. Most reviews (n=31) included primary studies focused on both men and women, who were mostly adults (vs youth under age 24 years). For less than half of the reviews (46%), it was the case that all or most primary studies (as opposed to few or none) collected data with men/boys.

The most common types of interventions described were (in order): community-based education and service delivery approaches, facility-based education and service delivery approaches, couples counselling, reaching men via their partners, mass media campaigns, school-based programmes and ‘gender transformative’ workshops. According to the WHO, gender transformative programmes/approaches address the causes of gender-based health inequities by including ways to transform harmful gender norms, roles and relations.^28^

Finally, funding for the reviews was mentioned by about two-thirds (66%) of reviews, with the most common sources of funding being the National Institutes of Health, the Bill & Melinda Gates Foundation, United States Agency for International Development/U.S. President’s Emergency Plan for AIDS Relief and WHO (each at about 10–15%).

### Quality and risk of bias of the reviews

Detailed quality assessment findings are included in table 3. About two-thirds (66%) of the 35 reviews were categorised as high quality. The remaining 34% were found to be moderate quality. In addition, all six reviews that included meta-analyses were found to have used appropriate methods for statistical combination of results. Criteria with the most room for improvement included accounting for quality/risk of bias when interpreting results (with 57% of reviews meeting this criteria), having a pre-established review protocol (63%), having more than one reviewer extract data (66%), and discussing heterogeneity of results and possible reasons for it (71%). There were no discernible patterns of quality/risk of bias across reviews falling under men as clients/partners/agents of change nor SRHR areas.

**Table 3:** Quality assessment findings, per AMSTAR 2 tool

Quality criteria	# of reviews meeting criteria, out of 35 total	%
1. Inclusion criteria covered PICO components	32	92%
2. Review protocol pre-established	22 (of which nine only partially met criteria)	63%
3. Described study design selection	33	94%
4. Used comprehensive literature search strategy	29	83%
5. Study selection performed by multiple reviewers	27	77%
6. Data extraction performed by multiple reviewers	23	66%
7. Described studies in adequate detail (eg, populations, interventions, comparators, outcomes, research designs)	35 (of which 16 only partially met criteria)	100%
8. Assessed study quality/risk of bias in a comprehensive manner (ideally using validated tool)	27 (of which six only partially met criteria)	77%
9. Accounted for study quality/risk of bias in interpretation of results	20	57%
10. Discussed heterogeneity of results and possible reasons for it	25	71%
11. Reported whether any conflicts of interest and any sources of funding for the review	32	91%
# of criteria met*	Out of n=31 reviews:	
High quality: met 9–11 criteria	23 *(6 met all 11 criteria*)	66%
Moderate quality: met 6–8 criteria	12	34%
*If meta-analysis*, used appropriate methods for the statistical combination of results†	6 out of 6 reviews including meta-analyses	100%

* Note that we dropped five studies due to poor quality (score of ≤5), reducing the number of included studies from 40 to 35.

† Appropriate methods for the statistical combination of results: justified combining data via meta-analysis; used weighting technique to combine study results; accounted for heterogeneity between studies; separated findings by randomizedrandomised controlled trials versus non-randomizedrandomised designs.

AMSTAR 2A Measurement Tool to Assess Systematic Reviews, version 2PICOPopulation, Intervention, Comparator group, Outcome

There was a relatively small degree of overlap in primary papers across reviews (114 of the 1080 total were included in at least two reviews).

### Engaging men and boys as clients

Thirteen (13) systematic reviews were included under the men as clients category.^29^,^41^ Specific areas of focus included HIV testing (3); voluntary medical male circumcision (3); linkage to/retention in HIV care (2); PrEP (1); HIV services in general (2); men as family planning/contraceptive users (2); and HIV-focused sexual risk reduction for adolescent boys and young men (1). All 13 included primary papers from Africa (11 focused on that region by design); two also included Latin America and the Caribbean, and one Europe. The most common types of interventions assessed in reviews were community service delivery approaches (92% of the reviews), health facility service delivery approaches (92%), community education (77%), reaching men via their partners (53%), health facility education (46%) and mass media social mobilisation campaigns (46%).

#### Quality of the evidence

Most reviews (but not all) found an adequate volume of evidence and these consistently reported this evidence was high quality. However, reviews also often highlighted a need for more consistency of study designs and outcome measures to improve comparability. Several reviews concluded there was an insufficient volume of evidence in certain important and timely areas—such as reviews of men as family planning users,^31 42^ peer support as a strategy or reviews that addressed sexually transmitted infections beyond HIV,^41^ PrEP^40 41^ or HIV self-testing (a more recent innovation).^37 38^

#### Intervention effectiveness

Overall, reviews of interventions seeking to increase men’s and boys’ use of HIV testing and VMMC—the bulk of reviews in this section—reported high levels of effectiveness across the types of strategies employed. For HIV testing, community-based (vs facility-based) service deliveries (eg, at mobile units; community venues; homes) were the most prevalent and effective method for improving testing uptake among men.^33 41^ This was attributed to improved convenience (especially related to avoiding long wait times) and privacy (especially to avoid perceived stigma associated with going to a health facility, which is also often dominated by women). Relatedly, reviews also highlighted the need to promote welcoming health facility environments for men seeking services. HIV self-testing was perceived to further increase convenience and privacy, as well as perceived autonomy around testing,^33 34 37 38 41^ although linkage to care in case of a positive HIV test result may be more difficult than health worker-administered testing.^38^ It was also clear from the reviews that men wanted HIV self-tests to be free or very low-cost.

Regarding VMMC, reviews found that community and school campaigns were effective at improving uptake among adolescent boys (usually defined in reviews as ages 10–19 years), while mobile services, home-based testing with active referral and follow-up, and routine facility service delivery were more likely to reach older men.^35^ A need was noted for improved tailoring of demand creation and patient education to different age groups of men—especially for adolescents specifically (including those who are not yet sexually active)—and accounting for whether the context was one where traditional circumcision was prevalent or not.^29 35^ Reviews also concluded that men and boys routinely prefer incentives for VMMC, which they see as fair compensation.^32^

Community-based service delivery was also found to be more preferred and effective than facility-based services, for PrEP initiation among men.^40^ As for other HIV services, to be effective, such approaches require person-centred services and non-judgmental healthcare workers.

The two reviews focusing on linkage to/retention in HIV care^36 38^ showed substantial room for improvement. For instance, a review of linkage to care of men newly testing positive via HIV self-testing found variable and consistently suboptimal linkage to care, with the highest rate being 72%.^38^ A review of interventions for retention in HIV care found men were substantially less likely than women to remain in care, with less than half of men on average retained at 24 months.^36^ Psychosocial vulnerabilities, often associated with harmful masculinities (eg, shame, loss of dignity), appeared to be key barriers to men’s engagement in care. Most evidence of effective interventions was for psychosocial support groups for people living with HIV, but these were rarely tailored for men specifically—a key gap highlighted by reviews.^36^ Other promising solutions in need of further evaluation include peer encouragement improved use of mobile technologies, financial incentives and community-based antiretroviral therapy initiation/distribution.

More work is also needed to engage men as family planning/contraceptive *users* (vs partners of women as family planning users), with reviews noting that studies consistently find that men want information on this topic.^31 42 43^ Reviews also called for greater attention to developing and promoting new family planning methods for men, noting that the only male methods currently available are vasectomy, male/external condoms and withdrawal.^31^ Overall, these reviews concluded that what is required is a fundamental re-orientation of the field to see men as having rights to information and services as women do, including better integrating men into national family planning agendas.^30 31^

The paper reviewing interventions to reduce HIV/sexual risk among adolescent boys and young men found that these interventions have consistently demonstrated positive effects on outcomes like condom use, reduction of multiple sexual partners, abstinence, alcohol and substance abuse and HIV testing.^39^ Both school-based and community-based approaches were found to be important. In-school programming capitalises on comprehensive sexuality education already happening (increasing feasibility and cost-effectiveness), while programming for out-of-school youth may reach more vulnerable individuals. Mixed-gender sessions, with opportunities for single-sex sessions, were recommended.

Across the reviews, authors consistently concluded that multilevel, multicomponent interventions to increase men’s use of SRH services were more effective than stand-alone/unidimensional interventions. Multicomponent interventions often entailed generating informed demand for the service (eg, via community education and providing comprehensive information to clients), improving supply (eg, offering community-based services; male-friendly health services) and creating an enabling environment for men’s uptake of SRH services to ensure men know about the service/product and do not feel shame when accessing services (normative, policy).

Additional recommendations from reviews included explicitly addressing gender norms to increase men’s use of SRH services/products and the importance of fully informing men to promote informed choice and reduce potential harms. For instance, fully informing men about HIV self-testing includes offering supportive messaging about the process of confirmatory testing/linkage to care^33 37 38^; for VMMC, improving understanding of risk reduction messaging especially among adolescent boys^29^; and for vasectomy informing men that this is a permanent method in contexts with limited availability of reversal services.^30^

#### Future focus for interventions and research

Finally, most reviews recommended that, given extant evidence of effectiveness, future efforts focus on increasing the scale at which services and interventions to increase men and boy’s SRH service uptake are delivered. Moreover, while interventions were successful overall, it is often not clear how true this was for men at highest risk of poor SRHR outcomes for themselves and/or their partners/families, who are often not easily identifiable and may also be hardest to reach.^1 20 35^ Several reviews concluded that context-specific strategies designed via formative research are likely required.^35 37^ There is also a need for more formative research to inform tailoring of interventions and messaging to men (vs women, eg, for HIV support groups) and different ages of men (eg, for VMMC). Additionally, reviews recommended more comparable evaluation designs and outcome measures and more disaggregation of data/results by gender, age group and level of risk/vulnerability.

### Engaging men as partners

Twelve (12) systematic reviews were included under the men as partners category.^42^,^53^ Specific areas of focus included prevention of mother-to-child transmission (PMTCT) of HIV (5), couples HIV testing (1), HIV risk reduction among sero-discordant couples (1), maternal and newborn health (3), family planning (1) and reproductive health (1). All 12 included primary papers from Africa (seven focused on that region by design); one review also included Asia and Latin America and the Caribbean, another Northern America and another Asia. The most common types of interventions assessed in reviews were couples counselling (100% of reviews), health facility education (92%), community education (83%), reaching men via their partners (83%), and health facility service delivery approaches (58%).

#### Quality of the evidence

Most reviews in this section reported variable quality of studies/degrees of risk of bias, and several reported low quality/high risk of bias. The main identified issue was a lack of randomised controlled trials or experimental studies more broadly. Many studies, particularly those focused on PMTCT and maternal and newborn health, also did not conduct research with men directly but only their female partners.

#### Intervention effectiveness

Overall, reviews found that men want to be involved as partners to improve SRHR and that male partner involvement is highly beneficial to a range of outcomes (HIV transmission, maternal and newborn health, reproductive health, contraceptive uptake and use) and is not detrimental to women. One review of eight different approaches to PMTCT found that male partner involvement was one of only two effective approaches.^44^ A meta-analysis of the impact of 17 male involvement PMTCT interventions found a statistically significant increase in maternal antiretroviral therapy uptake, the main outcome of interest.^45^

All reviews included couples counselling approaches, for instance, related to preventing HIV transmission, maternal and newborn health, and reproductive health, and consistently found it to be effective as long as counsellors were specially trained, perceived by clients to be highly acceptable (eg, community health workers; HIV-positive counsellors for PMTCT), and well-established behavioural techniques are used (eg, cognitive behavioural skills). Couples HIV counselling and testing specifically is an important option within a range of testing options, especially for pregnant women and their partners, even if less than half of couples offered it will test as a couple.^51^ Uptake of couples HIV testing was found to increase when one member of the couple tests independently first.^51^ Ensuring health facility environments and staff are welcoming when men seek to accompany their partner that was also consistently highlighted as an important approach across reviews.

Reviews also emphasised the importance of interventions conveying detailed knowledge among men, for example, about the elements of the PMTCT cascade (eg, HIV testing during pregnancy, uptake and adherence to antiretroviral therapy, facility delivery, sate infant feeding practices, and infant HIV prophylaxis and testing), what to do in case of birth complications/obstetric emergencies and how to safely conceive if one/both members of couple is living with HIV. Relatedly, many reviews stressed a need for interventions to more clearly define ‘behavioural asks’ of their interventions, regarding how exactly men should be involved, for example, within the PMTCT cascade or preventing obstetric emergencies.

Reviews did not find evidence of harmful effects of male involvement on women’s SRHR outcomes, but also stressed the importance of careful design of interventions and content to ensure avoidance of harms. This is especially true for certain populations, for instance, HIV sero-discordant couples, who often face a range of challenges (HIV status disclosure, communication dynamics, balancing desire for fertility and reproduction).^47^ For maternal and newborn health interventions in particular, it may be especially critical to avoid the risk of male involvement disempowering women in one of few domains in which they are empowered in many patriarchal societies.^48^

Most reviews, including several meta-analyses, concluded that interventions with multiple integrated components to engage male partners, along with other healthcare delivery and community-based approaches, are more effective than ‘single’, ‘one-off’ interventions. For instance, PMTCT invitation letters alone (ie, letters from the health facility inviting men to attend, often delivered by their female partners) were found to be ineffective.^45 49^ An example of a multicomponent programme would be couples counselling plus a welcoming health facility plus activities to normalise men’s involvement in their partners’ SRHR. Several reviews highlighted the importance of incorporating gender-transformative conceptual approaches into male involvement intervention design, including gendered relationship dynamics.^42 48 53^ Several reviews of male involvement in family planning/reproductive health stressed the importance of national SRHR programmes including men as allies and equal partners rather than considering their presence a barrier to effective programme delivery, based on its findings that all types of male involvement strategies were effective in engaging men and improving family planning and reproductive health outcomes.^42 43^

#### Future focus for interventions and research

In general, reviews indicated that that there is now sufficient evidence documenting benefits and low risks overall of male partner involvement. Yet, they also concluded that there is a need for more studies that rigorously evaluate male engagement strategies, especially via randomised controlled trials, and ideally with the inclusion of multicomponent strategies. One review highlighted the need to expand the outcomes measured, such as ‘met need for family planning.’^42^ Several reviews noted the importance of careful documentation of any risks and unintended consequences of male partner involvement interventions. Finally, reviews consistently highlighted the need to conduct research with both women and men/their partners.

### Engaging men and boys as agents of change/promoting an enabling environment for sexual and reproductive health and rights (SRHR)

Ten (10) systematic reviews were included under the men as agents of change category.^54^,^63^ Specific areas of focus included intimate partner violence (IPV)/violence against women and girls (VAWG)/GBV (7), gender norms/relations (2), GBV and HIV (1), and SRHR (1). Henceforth, we will refer to IPV, VAWG and GBV collectively as GBV. All 10 reviews included primary papers from Africa (one focused on that region by design); nine included Asia, seven Latin America and the Caribbean, four Northern America, three Europe and one Oceania. The most common types of interventions assessed in reviews were gender transformative workshops (90% of reviews), community education (70%), structural interventions (70%), school-based programmes (60%) and mass media social mobilisation campaigns (50%).

#### Quality of the evidence

Varied quality of reviews was reported. The rigour of study designs appears to have improved over time, but in some cases, certain reviews appear to have applied more stringent quality assessments than others, leading to low quality ratings even among reviews that mostly included randomised controlled trials due to aspects like participant selection, randomisation and deviations from the intended intervention.^56 59^ However, these reviews also conceded that these are complex interventions implemented in complex environments, and hence ‘…potentially promising conclusions from implementing well-planned interventions in complex environments should not be ignored’.^56^

#### Intervention effectiveness

There is substantial evidence for gender transformative programmes to engage men in order to reduce GBV and improve SRHR and gender relations. A meta-analysis found that overall, community-level or group-based interventions significantly reduced experience of physical IPV (adjusted OR (aOR) 0.79, 95% CI 0.65 to 0.96); experience of sexual IPV (aOR 0.80, 95% CI 0.67 to 0.95); experience of emotional IPV (aOR=0.81, 95% CI 0.69 to 0.95); and perpetration of sexual IPV (aOR 0.82, 95% CI 0.68 to 0.98).^63^ Another review found that 61/68 studies reviewed showed evidence of efficacy in relation to behavioural or attitudinal outcomes,^56^ and another found 74% of evaluations with positive impact on gender- or health-related indicators.^57^ The field has evolved from demonstrating that it is possible to prevent GBV, including by engaging men,^55^ to determining best models/best practices for doing so.^56 60^ Reviews that covered interventions to engage men and boys as one of several approaches to address GBV found that interventions that engaged men and boys were more effective than those that did not.^57 62^ Gender transformative interventions typically focused on more than one SRHR component—most commonly GBV and sexual health/HIV. They consistently led to improvement in important outcomes along the pathway to improved SRHR outcomes, including reducing GBV, for example, attitudes toward violence, communication within couples and families, conflict resolution skills, bystander behaviours and shared housework.

The reviews also did not report that any interventions led to negative effects on prevalence of SRHR outcomes (eg, GBV perpetration). Still, most reviews stressed the importance of careful tracking of any potential harms/unintended consequences. Notably, one systematic review related to IPV prevention did refer to several primary studies relating qualitative reports of unintended consequences (ie, more violent/controlling behaviours of male partners).^54^

Several specific gender transformative interventions have now been evaluated and consistently found to be effective, including via studies based in multiple low- and middle-income countries. These include Stepping Stones, SASA!, Program H, C-Change and the Male Norms Initiative. Similar implementation best practices for such interventions have emerged across reviews, including: (1) multicomponent activities to educate, persuade, model and enable positive behaviour change; (2) sessions that are participatory, promote critical reflection around gender roles and build couple communication and conflict resolution skills; (3) multilevel programming that reaches beyond target groups to mobilise the wider community in adopting egalitarian gender norms and practices; (4) careful selection and training of facilitators; (5) sufficient duration (often described as ≥3 months^56^; ≥8 sessions^59^) as shorter programmes were less/not effective^56 59 62 63^; and (6) working with both women/girls *and* men/boys, either in mixed sex groups or separately (and ideally both). Combining genders in the same group is important to reflect and interact together, while it is also important to include opportunities for single-sex-only groups — for example, when introducing new sensitive topics (to discuss separately before coming together), and to give men/boys opportunities to bond and discuss difficult topics openly with other men/boys. Several reviews also noted the importance of the sociocultural contextualization of intervention design via involving communities in the development phase, to increase acceptability, impact and sustainability.^58 59^

A review focused on mHealth and gender relations found that mHealth interventions, which tended to be simple SMS-based approaches focused on promoting specific behaviours (most re: maternal and newborn health or HIV care reminders), positively affected gender relations in multiple ways.^61^ For instance, they enhanced partner communication, fostered emotional support and increased detailed knowledge. This review also noted the importance of careful design of mHealth interventions to explicitly take into account and seek to transform gender norms/relations, and to avoid harms.

#### Future focus for interventions and research

Most reviews stressed the need for structural change alongside current programming, highlighting, for example, that few interventions have gone beyond small group or community level. Specific examples include social protection/poverty reduction and improving the policy/legal environment in low- and middle-income countries to counter GBV (legislation re: domestic violence is common but is rarely adequately implemented).^55^ Review authors consistently highlighted the continued need for further refining understanding of the most crucial components within complex multilevel, multicomponent interventions, to increase impact but also scalability (including via information about cost-effectiveness). Reviews also routinely recommended more qualitative research to unpack intervention mechanisms of change/causal pathways and to identify any harms/unintended consequences. Finally, a number of reviews noted that to date, gender transformative programmes have been highly heteronormative and recommended that programing with men and boys more intentionally address homophobia, examine gender diversity and promote SRHR for LGBTQ communities.^56 57^

### Conceptual framework

**Figure 2:**
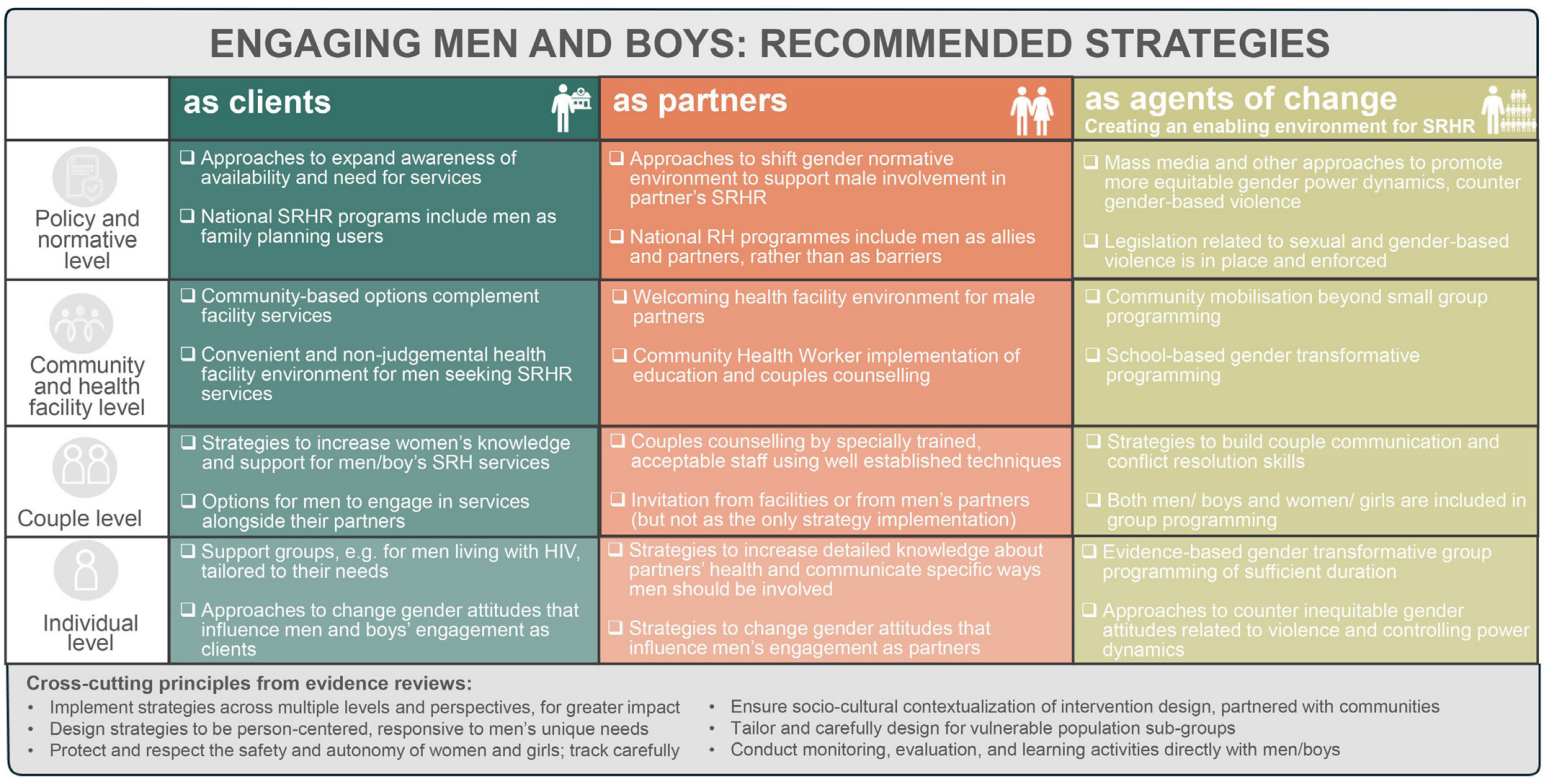
Conceptual framework of recommended strategies for engaging men and boys to improve SRHR outcomes. RH, reproductive health; SRH, sexual and reproductive health; SRHR, sexual and reproductive health and rights.

Figure 2 presents a summative conceptual framework delineating types of male engagement interventions most consistently found to be effective in our systematic review of reviews on male engagement. It details specific activities related to men as clients, as partners or as agents of change/on creating an enabling environment for the promotion of SRHR. These interventions are positioned at different levels of the social ecological framework (eg, individual, couple, community and health facility, and policy and normative).^26^ Additionally, this figure emphasises the importance of implementing strategies across multiple levels and perspectives, for greater impact on SRHR outcomes. It also highlights key guiding principles, for example, designing strategies to be person-centred and responsive of men’s unique needs. It also provides potential guidance around what a comprehensive national effort to promote male engagement in SRHR could look like.

## Discussion

In this systematic review of reviews, we sought to bring together the evidence around interventions to engage men and boys to improve SRHR in low- and middle-income countries. Across 35 systematic reviews of ~960 primary studies, reviews consistently concluded that men are willing to engage as clients, as partners and as agents of change and that male engagement was measurably beneficial to both women and men’s SRHR outcomes. Effective intervention strategies were identified for each of the SRHR topic areas that reviews focused on, including HIV testing, VMMC, HIV risk reduction, family planning, PMTCT, maternal and newborn health, reproductive health and GBV. To engage men and boys as clients, effective strategies focus on providing convenient and private services/product options, such as community-based HIV services, and increasing awareness and demand,^64^ as well as reaching/expanding access to men as family planning users. To engage men as partners, effective strategies concentrate on offering couples counselling by specially trained staff using evidence-based counselling techniques, ensuring men know precisely how they need to be involved and avoiding only trying to reach men secondhand such as via their partners. To engage men as agents of change, gender transformative programming at the group and community levels has consistently proven effective—with multiple programmes now evaluated across contexts and with specific best practices identified for implementation—especially when complemented with community mobilisation to shift gender norms. These interventions can be further tailored to new social-cultural contexts via community-partnered formative assessments to maximise acceptability, resonance, impact, and sustainability and minimise any potential unintended harms.

None of the 35 included reviews found any primary study showing adverse impacts of an intervention on prevalence of women’s SRHR outcomes, such as increasing GBV perpetration or victimisation, or impeding women’s access to SRHR services. Most, although not all, reviews appeared to have assessed adverse effects reported in primary studies, although this was rarely a primary objective. Nonetheless, reviews consistently emphasised the importance of careful intervention design in partnership with communities, and monitoring of any harms, especially in the case of vulnerable subpopulations.

Nearly all reviews found that multicomponent and multilevel interventions were most effective. Examples include complementing individual-level education and service delivery with promoting society-wide awareness of HIV prevention products/diagnostics or family planning products or complementing couples counselling with making health facilities more welcoming towards men and changing the normative environment to foster men’s support of their partners’ SRHR. Moreover, there is a clear need for structural change, most critically a shift in the gender norms and systems that sustain gender inequality and violence.^65^ These conclusions align with findings from another systematic review of reviews which sought to gauge the proportion of SRHR programmes for men and boys that met the definition of gender transformative and found that only a small proportion (<10%) did so.^12^

There was also notable consistency in specific successful approaches found across reviews. One such approach was changing gender attitudes and norms. Currently, this is seen mainly in GBV prevention programmes (and sometimes HIV programmes)—other SRHR domains would benefit from this approach such as male infertility or sexually transmitted infections beyond HIV. A second approach was building couple communication and conflict resolution skills as a key facilitator of a range of men’s and couples’ SRHR outcomes. A third was promoting men’s detailed knowledge of products/services. There is notably low population-level awareness of certain products/services, like vasectomy and HIV self-testing, as well as specific ways men should support women’s SRHR. It is the responsibility of public health systems, not men themselves, to raise this awareness and promote informed decision-making.

The quality of the included reviews was quite high, per the AMSTAR 2 tool criteria. Review findings varied regarding quality/risk of bias for the primary studies they included. For the most part, reviews under men as clients were high quality, those under men as partners were quite low quality (particularly for reviews related to PMTCT and maternal and newborn health), and under men as agents of change were variable quality (with reviews appearing to differ in the standards applied). A critical quandary for the field is how best to evaluate, what standard of evidence should be required, for complex, multicomponent, multi-level interventions that are often implemented in complex environments.

There was a heavy geographic focus on Africa, and specifically Eastern and Southern Africa (sometimes by design, sometimes because primary studies had only/mainly been focused in that region). This makes our findings most representative of, and generalisable to, that region. This is especially true for reviews under the men as clients and men as partners categories—while reviews under men as agents of change tended to focus much more broadly across geographic regions. There is a need for geographical expansion of the evidence base for some of this work beyond Eastern and Southern Africa, while the evidence for men as agents of change is relatively global at this point.

There were also several gaps regarding the seven SHRH domains and types of interventions. Very few reviews covered the WHO SRHR domains of preventing unsafe abortions or SRHR in disease outbreaks. A limited number of reviews focused on men as contraceptive *users* (vs only as partners),^30 31 42^ with each concluding that there is a need for expanded research on this topic, particularly as new male contraceptive methods become available. Only two reviews^40 42^ directly addressed the critical HIV prevention option of PrEP—with men as PrEP users or partners of users. No reviews focused explicitly on sexually transmitted infections besides HIV. In addition, no reviews were available that focused on sexual and gender-based violence as experienced by men and boys—which evidence has shown is quite common and associated with various negative health outcomes for them and their partners.^4 5^ With regard to types of interventions, virtually no approaches to engaging men included social media (noting the review related to mHealth primary focused on SMS text/voice messages)—which may be an important future focus since many reviews highlighted a need to promote broad societal awareness of SRHR services/products as well as shift social norms. Almost no reviews included interventions delivered at workplaces (and the two that did focused on HIV service delivery only).

As suspected when taking on this review, there was substantial siloing by SRHR domain/topic of evidence reviewed. Over half (54%) of reviews covered only one domain, and most others covered two domains. Moreover, most reviewers (though not all) reflected on and compared their findings only with other systematic reviews in their own domain or even their own particular area such as couples HIV testing or PMTCT. Our review of reviews clarified and confirmed that there are markedly similar findings across SRHR topic areas reaffirming men’s willingness to engage, and their desire for information as well as few concerns about potential harms for women.

It is important to consider our findings in light of several potential limitations. Findings from a systematic review of reviews are necessarily reflective of the set of systematic reviews available for inclusion. For instance, there was a heavy focus on HIV services in our review, given so many included reviews focused on this topic. The magnitude and breadth of included reviews help assuage this concern. In addition, conducting a review of reviews, rather than of primary papers, may have led to the exclusion of relevant primary papers. Cochrane guidelines advise against adding primary papers to the set of included systematic reviews in most cases.^8^ Limiting eligibility to reviews published in English could also have excluded reviews focusing more on certain regions (eg, West Africa; Latin America). Additionally, we did not include certain topics that from our perspective fell outside the scope of this review: men as caregivers (no explicit SRHR outcomes); couples/men’s infertility issues (available reviews were largely biomedical in nature); and child marriage (a separate and large body of research in itself). Finally, we did not search the ‘grey literature’ which may have resulted in missing certain reviews, although systematic reviews are typically published in peer-reviewed literature.

## Conclusion

We believe that given the substantial evidence supporting a range of successful interventions to engage men and boys to improve SRHR, the time has come to widely scale up these interventions. A combination of multiple strategies at different levels, integration across SRHR areas and with programming that also includes women and children and fundamentally transforms gender relations and systems,^12 65^ will be critical for achieving the adequate scale and sustainability required for public heath impact on SRHR outcomes. It is clear the field is still evolving and further research is warranted, including on effectiveness in certain geographic areas, optimal implementation models, new products/technologies, appropriate sociocultural contextualisation and continued monitoring for any harms. Yet, we now know enough to move ahead with scaled up programming—the need for improving SRHR among men, women, families and communities is too great to wait.

## supplementary material

10.1136/bmjopen-2024-083950 online supplemental file 1

10.1136/bmjopen-2024-083950 online supplemental file 2

## Data Availability

Data sharing not applicable as no datasets generated and/or analysed for this study.
