# Supplementary material for: Systematic review of reviews on interventions to engage men and boys as clients, partners and agents of change for improved sexual and reproductive health and rights
Source: BMJ Open. 2025 Jan 20;15(1):e083950. doi: 10.1136/bmjopen-2024-083950 (PMC11751930; doi:10.1136/bmjopen-2024-083950)
Supplement: online supplemental file 1 [file bmjopen-15-1-s001.docx]

**Supplemental File 1. Database search summary table**

| **Database** | **Date search conducted** | **Specified date range of search** | **Resulting number of citations** | **Search terms (same for both search timepoints)** |
| --- | --- | --- | --- | --- |
| **PubMed** | October 18 to November 9, 2022 | January 1, 2015 to November 8, 2022 | 70 | ("systematic*"[Title] AND "review"[Title]) OR "systematic overview*"[Title] OR "cochrane review*"[Title] OR "systemic review*"[Title] OR "scoping review"[Title] OR "scoping literature review"[Title] OR "mapping review"[Title] OR "umbrella review*"[Title] OR "review of reviews"[Title] OR "overview of reviews"[Title] OR "meta-review"[Title] OR "integrative review"[Title] OR "integrated review"[Title] OR "integrative overview"[Title] OR ("meta-synthesis"[Title] OR "metasynthesis"[Title]) OR ("quantitative review"[Title] OR "quantitative synthesis"[Title] OR "research synthesis"[Title] OR "meta-ethnography"[Title] OR "systematic literature search"[Title] OR "systematic literature research"[Title] OR "meta-analyses"[Title] OR "metaanalyses"[Title] OR "metaanalysis"[Title] OR "meta-analysis"[Title] OR "meta analytic review"[Title] OR "meta analytical review"[Title] OR "meta-analysis"[Title]) AND (Men[Text Word] OR Males[Text Word] OR Adolescent Boys[Text Word] OR Young Men [Text Word] OR ABYM[Text Word] OR adolescent boys[Text Word] OR young men[Text Word] OR heterosexual men[Text Word] OR men as partners [Text Word] OR male sexual partners [Text Word] OR male partners [Text Word] OR couples [Text Word] OR heterosexual couples [Text Word] OR men living with HIV [Text Word] OR youth [Text Word] OR adolescents [Text Word] OR very young adolescents [Text Word] OR sero-discordant [Text Word]) AND (Sexual and reproductive health [Text Word] OR SRH [Text Word] OR sexual and reproductive health and rights [Text Word] OR SRHR [Text Word] OR sexual health [Text Word] OR reproductive health OR HIV prevention [Text Word] OR HIV testing [Text Word] OR voluntary medical male circumcision [Text Word] OR VMMC [Text Word] OR HIV care[Text Word] OR HIV treatment[Text Word] OR sexually transmitted infection [Text Word] OR STI [Text Word] OR prevention of maternal to child transmission [Text Word] OR PMTCT [Text Word] OR family planning[Text Word] OR contracept[Text Word] OR abortion [Text Word] OR infertility [Text Word] OR reproductive cancers [Text Word] OR gender-based violence[Text Word] OR intimate partner violence[Text Word] OR domestic violence[Text Word] OR sexual violence[Text Word] OR violence against women[Text Word] OR violence against women and girls [Text Word] OR comprehensive sexuality education [Text Word] OR antenatal care [Text Word] OR childbirth [Text Word] OR postnatal care[Text Word] OR sexual health [Text Word]) AND (program*[Title/Abstract] OR intervention [Title/Abstract] OR service uptake [Title/Abstract] OR service delivery [Title/Abstract] OR service use [Title/Abstract]) AND (Male engagement[Text Word] OR Engaging men[Text Word] OR Male involvement[Text Word] OR Involving men[Title/Abstract] OR Gender roles[Text Word] OR gender norms[Text Word] OR social norms[Text Word] OR masculinit*[Text Word] OR gender transformative[Text Word] OR gender-transformative[Text Word] OR transform gender norms[Text Word] OR gender attitudes[Text Word] OR couples-based[Text Word] OR couples-focused[Text Word] OR partner communication[Text Word] OR partner support[Text Word] OR power[Text Word] OR gender integrat*[Text Word]) |
|  | September 9, 2024 | November 9, 2022 to December 31, 2023 | 28 |  |
| **CINAHL** | October 18 to November 9, 2022 | January 1, 2015 to November 8, 2022 | 291 | ( TI ( ("systematic" AND "review") OR "systematic overview*" OR "cochrane review*" OR "systemic review*" OR "scoping review" OR "scoping literature review" OR "mapping review" OR "umbrella review*" OR "review of reviews" OR "overview of reviews" OR "meta-review" OR "integrative review" OR "integrated review" OR "integrative overview" OR ("meta-synthesis" OR "metasynthesis") OR ("quantitative review" OR "quantitative synthesis" OR "research synthesis" OR "meta-ethnography" OR "systematic literature search" OR "systematic literature research" OR "meta-analyses" OR "metaanalyses" OR "metaanalysis" OR "meta-analysis" OR "meta analytic review" OR "meta analytical review" OR "meta-analysis") ) )  AND ( TX ( Men OR Males OR Adolescent Boys OR Young Men OR ABYM OR adolescent boys OR young men OR heterosexual men OR men as partners OR male sexual partners OR male partners OR couples OR heterosexual couple* OR men living with HIV OR youth OR adolescent* OR very young adolescents OR sero-discordant ) )  AND ( TX ( Sexual and reproductive health OR SRH OR sexual and reproductive health and rights OR SRHR OR sexual health OR reproductive health OR HIV prevention OR HIV testing OR voluntary medical male circumcision OR VMMC OR HIV care OR HIV treatment OR sexually transmitted infection OR STI OR prevention of maternal to child transmission OR PMTCT OR family planning OR contracept OR abortion OR infertility OR reproductive cancers OR gender-based violence OR intimate partner violence OR domestic violence OR sexual violence OR violence against women OR violence against women and girls OR comprehensive sexuality education OR antenatal care OR childbirth OR postnatal care OR sexual health ) )  AND ( ( program* OR intervention OR service uptake OR service delivery OR service use ) )  AND ( TX ( Male engagement OR Engaging men OR Male involvement OR Involving men OR Gender roles OR gender norms OR social norms OR masculinit* OR gender transformative OR gender-transformative OR transform gender norms OR gender attitudes OR couples-based OR couples-focused OR partner communication OR partner support OR power OR gender integrat* ) ) |
|  | September 9, 2024 | November 9, 2022 to December 31, 2023 | 43 |  |
| **Web of Science** | October 18 to November 9, 2022 | January 1, 2015 to November 8, 2022 | 225 | ((((TI=(("systematic" AND "review") OR "systematic overview*" OR "cochrane review*" OR "systemic review*" OR "scoping review" OR "scoping literature review" OR "mapping review" OR "umbrella review*" OR "review of reviews" OR "overview of reviews" OR "meta-review" OR "integrative review" OR "integrated review" OR "integrative overview" OR ("meta-synthesis" OR "metasynthesis") OR ("quantitative review" OR "quantitative synthesis" OR "research synthesis" OR "meta-ethnography" OR "systematic literature search" OR "systematic literature research" OR "meta-analyses" OR "metaanalysis" OR "metaanalysis" OR "meta-analysis" OR "meta analytic review" OR "meta analytical review" OR "meta-analysis")))  AND AB=(Men OR Males OR Adolescent Boys OR Young Men OR ABYM OR adolescent boys OR young men OR heterosexual men OR men as partners OR male sexual partners OR male partners OR couples OR heterosexual couple* OR men living with HIV OR youth OR adolescent* OR very young adolescents OR sero-discordant))  AND AB=(Sexual and reproductive health OR SRH OR sexual and reproductive health and rights OR SRHR OR sexual health OR reproductive health OR HIV prevention OR HIV testing OR voluntary medical male circumcision OR VMMC OR HIV care OR HIV treatment OR sexually transmitted infection OR STI OR prevention of maternal to child transmission OR PMTCT OR family planning OR contracept OR abortion OR infertility OR reproductive cancers OR gender-based violence OR intimate partner violence OR domestic violence OR sexual violence OR violence against women OR violence against women and girls OR comprehensive sexuality education OR antenatal care OR childbirth OR postnatal care OR sexual health))  AND AB=(program* OR intervention OR service uptake OR service delivery OR service use))  AND AB=(Male engagement OR Engaging men OR Male involvement OR Involving men OR Gender roles OR gender norms OR social norms OR masculinit* OR gender transformative OR gender-transformative OR transform gender norms OR gender attitudes OR couples-based OR couples-focused OR partner communication OR partner support OR power OR gender integrat*) |
|  | September 9, 2024 | November 9, 2022 to December 31, 2023 | 71 |  |
| **Cochrane Database of Systematic Reviews** | October 18 to November 9, 2022 | January 1, 2015 to November 8, 2022 | 165 | (("systematic" AND "review") OR systematic NEXT overview* OR cochrane NEXT review* OR systemic NEXT review* OR "scoping review" OR "scoping literature review" OR "mapping review" OR umbrella NEXT review* OR "review of reviews" OR "overview of reviews" OR "meta-review" OR "integrative review" OR "integrated review" OR "integrative overview" OR ("meta-synthesis" OR "metasynthesis") OR ("quantitative review" OR "quantitative synthesis" OR "research synthesis" OR "meta-ethnography" OR "systematic literature search" OR "systematic literature research" OR "meta-analyses" OR "metaanalyses" OR "metaanalysis" OR "meta-analysis" OR "meta analytic review" OR "meta analytical review" OR "meta-analysis"))  AND  (Men OR Males OR Adolescent Boys OR Young Men OR ABYM OR adolescent boys OR young men OR heterosexual men OR men as partners OR male sexual partners OR male partners OR couples OR heterosexual couple* OR men living with HIV OR youth OR adolescent* OR very young adolescents OR sero-discordant) in Title Abstract Keyword  AND  (Sexual and reproductive health OR SRH OR sexual and reproductive health and rights OR SRHR OR sexual health OR reproductive health OR HIV prevention OR HIV testing OR voluntary medical male circumcision OR VMMC OR HIV care OR HIV treatment OR sexually transmitted infection OR STI OR prevention of maternal to child transmission OR PMTCT OR family planning OR contracept OR abortion OR infertility OR reproductive cancers OR gender-based violence OR intimate partner violence OR domestic violence OR sexual violence OR violence against women OR violence against women and girls OR comprehensive sexuality education OR antenatal care OR childbirth OR postnatal care OR sexual health) in Title Abstract Keyword  AND  (program* OR intervention OR service uptake OR service delivery OR service use) in Title Abstract Keyword  AND (Male engagement OR Engaging men OR Male involvement OR Involving men OR Gender roles OR gender norms OR social norms OR masculinit* OR gender transformative OR gender-transformative OR transform gender norms OR gender attitudes OR couples-based OR couples-focused OR partner communication OR partner support OR power OR gender integrat*) in Title Abstract Keyword - (Word variations have been searched) |
|  | September 9, 2024 | November 9, 2022 to December 31, 2023 | 10 |  |
