# Supplementary material for: Systematic review of reviews on interventions to engage men and boys as clients, partners and agents of change for improved sexual and reproductive health and rights
Source: BMJ Open. 2025 Jan 20;15(1):e083950. doi: 10.1136/bmjopen-2024-083950 (PMC11751930; doi:10.1136/bmjopen-2024-083950)
Supplement: online supplemental file 2 [file bmjopen-15-1-s002.docx]

**Supplemental Table 1. Reviews excluded at or after the full text review stage, and main reason for exclusion (n=58)**

| **Author(s), year & Journal** | **Title** | **Main reason for exclusion** |
| --- | --- | --- |
| **Articles excluded at full text review stage** | | |
| Aibangbee et al. 2023  *International Journal of Public Health* | Migrant and refugee youth’s sexual  and reproductive health and rights: a  scoping review to inform policies and  programs | Outside of geographic focus^a^ |
| Alexander et al. 2023  *Curr HIV/AIDS Rep* | intervention approaches to address intimate partner violence and HIV: a scoping review of recent research | Doesn’t focus specifically on men and boys/male engagement |
| Amoo et al. 2019  *Cogent Social Sciences* | Are there traditional practices that affect men’s reproductive health in sub-Saharan Africa? A systematic review and meta-analysis approach | Doesn’t focus on reviewing interventions |
| Aung et al. 2020  *Global Health Science and Practice* | Effectiveness of mHealth interventions for improving contraceptive use in low- and middle-income countries: A systematic review | Doesn’t focus specifically on men and boys/male engagement |
| Beckham et al. 2016  *Journal of the International AIDS Society* | Marked sex differences in all-cause mortality on antiretroviral therapy in low- and middle-income countries: A systematic review and meta-analysis | Doesn’t focus on reviewing interventions |
| Casey et al. 2022  *Trauma, Violence, & Abuse* | Life course and socioecological influences on gender-equitable attitudes among men: A scoping review | Doesn’t focus on reviewing interventions |
| Caruso 2020  *Calidad de Vida y Salud* | Domestic and gender violence. Systematic review study intervention on the men involved | Full text not in English |
| Crepaz et al. 2015  *AIDS Care* | Are couple-based interventions more effective than interventions delivered to individuals in promoting HIV protective behaviors? A meta-analysis | Doesn’t focus specifically on men and boys/male engagement |
| Chiang et al. 2022  *Birth* | Men's experiences of antenatal care services in low-income and middle-income countries: A qualitative systematic review | Doesn’t focus on reviewing interventions |
| Ciocanel et al. 2017  *Journal of Youth and Adolescence* | Effectiveness of positive youth development interventions: A meta-analysis of randomized controlled trials | Outside of geographic focus^a^ |
| Colvin 2019  *The Lancet HIV* | Strategies for engaging men in HIV services | Not a systematic review ^b^ |
| Cuco et al. 2015  *SAHARA-J: Journal of Social Aspects of HIV/AIDS* | Male partners' involvement in prevention of mother-to-child HIV transmission in sub-Saharan Africa: A systematic review | Doesn’t focus on reviewing interventions |
| Condran et al. 2017  *Canadian Journal of Human Sexuality* | A scoping review of social media as a platform for multi-level sexual health promotion interventions | Outside of geographic focus^a^ |
| Davey et al. 2018  *AIDS and Behavior* | A systematic review of the current status of safer conception strategies for HIV affected heterosexual couples in sub-Saharan Africa | Doesn’t focus specifically on men and boys/male engagement |
| De Tomasi et al. 2019  *Medecine Et Sante Tropicales* | Good practices for retention in the circuit of prevention of Mother-Child Transmission of HIV in Sub-Saharan Africa: A systematic review of the literature | Full text not in English |
| Eqtait et al. 2019  *Open Journal of Nursing* | Male involvement in family planning: An integrative review | Doesn’t focus on reviewing interventions |
| Fu et al. 2022  *AIDS and Behavior* | Do couple-based interventions show larger effects in promoting HIV preventive behaviors than individualized interventions in couples? A systematic review and meta-analysis of 11 randomized controlled trials | Outside of geographic focus^a^ |
| Galle et al. 2021  *BMJ Global Health* | Systematic review of the concept "male involvement in maternal health" by natural language processing and descriptive analysis | Doesn’t focus on reviewing interventions |
| Gause et al. 2018  *Journal of Behavioral Medicine* | Meta-analyses of HIV prevention interventions targeting improved partner communication: Effects on partner communication and condom use frequency outcomes | Outside of geographic focus^a^ |
| Gebremeskel et al. 2021  *Biomed Research International* | Sex differences in HIV testing among older adults in sub-Saharan Africa: A systematic review | Doesn’t focus on reviewing interventions |
| Gerrits et al. 2023  *Sexual and Reproductive Health Matters* | Breaking the silence around infertility: a scoping review of interventions addressing infertility-related gendered stigmatisation in low- and middle-income countries | Doesn’t focus specifically on men and boys/male engagement |
| Gibbs 2017  *Global Health Action* | A global comprehensive review of economic interventions to prevent intimate partner violence and HIV risk behaviours | Doesn’t focus specifically on men and boys/male engagement |
| Graham et al. 2021  *Trauma, Violence, & Abuse* | Evaluations of prevention programs for sexual, dating, and intimate partner violence for boys and men: A systematic review | Outside of geographic focus^a^ |
| Gunawardena et al. 2019  *BMJ Global Health* | Predictors of pregnancy among young people in sub-Saharan Africa: A systematic review and narrative synthesis | Doesn’t focus on reviewing interventions |
| Hlongwa et al. 2020  *African Journal of AIDS Research* | Barriers to HIV testing uptake among men in sub-Saharan Africa: A scoping review | Doesn’t focus on reviewing interventions |
| Jewkes et al. 2015  *The Lancet* | From work with men and boys to changes of social norms and reduction of inequities in gender relations: A conceptual shift in prevention of violence against women and girls | Not a systematic review ^b^ |
| Kane et al. 2019  *Journal of adolescence* | Adolescent men's attitudes and decision making in relation to pregnancy and pregnancy outcomes: An integrative review of the literature from 2010 to 2017 | Outside of geographic focus^a^ |
| Kouta et al. 2015  *Health Education Research* | A systematic review of training interventions addressing sexual violence against marginalized at-risk groups of women | Outside of geographic focus^a^ |
| Ladur et al. 2021  *Midwifery* | Male involvement in promotion of safe motherhood in low- and middle-income countries: A scoping review | Doesn’t focus on reviewing interventions |
| Ma et al. 2019  *AIDS and Behavior* | Self-stigma reduction interventions for people living with HIV/AIDS and their families: A systematic review | Doesn’t focus specifically on men and boys/male engagement |
| Mantell et al. 2020  *Chapter in the book “Preventing HIV Among Young People in Southern and Eastern Africa: Emerging Evidence and Intervention Strategies”* | Are adolescent boys and young men being left behind? Missing discourse and missed opportunities for engagement in HIV prevention in Eastern and Southern Africa | Not a systematic review ^b^ |
| Muthoni et al. 2020  *AIDS and Behavior* | A systematic review of HIV interventions for young women in sub-Saharan Africa | Doesn’t focus specifically on men and boys/male engagement |
| Nguyen et al. 2023  *Advances in Dual Diagnosis* | Programme responses for men who perpetrate intimate partner violence in the context of alcohol or other drugs: a scoping review | Outside of geographic focus^a^ |
| Nickel et al. 2020  *Journal of Community Health* | Effectiveness of community-based health promotion interventions in urban areas: A systematic review | Outside of geographic focus^a^ |
| Ngidi et al. 2017  *African Journal Of Primary Health Care & Family Medicine* | Mapping evidence of interventions and strategies to bridge the gap in the implementation of the prevention of mother-to-child transmission of HIV programme policy in sub-Saharan countries: A scoping review | Doesn’t focus specifically on men and boys/male engagement |
| Park et al. 2023  *Psychology of Violence* | a systematic review and meta-analysis of bystander intervention programs for intimate partner violence and sexual assault | Outside of geographic focus^a^ |
| Sell et al. 2021  *Sexuality Research and Social Policy* | Comprehensive sex education addressing gender and power: A systematic review to investigate implementation and mechanisms of impact | Outside of geographic focus^a^ |
| Semahegn et al. 2019  *Reproductive Health* | Are interventions focused on gender-norms effective in preventing domestic violence against women in low and lower-middle income countries? A systematic review and meta-analysis | Doesn’t focus specifically on men and boys/male engagement |
| Shand 2021  *Oxford Research Encyclopedia of Global Public Health* | Engaging men in sexual and reproductive health | Not a systematic review ^b^ |
| Sharifipour, et al. 2022  *Health Promotion Perspectives* | Interventions to improve social support among postpartum mothers: A systematic review | Outside of geographic focus^a^ |
| Sharma et al. 2017  *PLOS Medicine* | Community-based strategies to strengthen men’s engagement in the HIV care cascade in sub-Saharan Africa | Not a systematic review ^b^ |
| Sharma et al. 2018  *BMC Pregnancy & Childbirth* | Systematic review of community participation interventions to improve maternal health outcomes in rural South Asia | Doesn’t focus specifically on men and boys/male engagement |
| Sileo et al. 2018  *AIDS and Behavior* | What role do masculine norms play in men's HIV testing in sub-Saharan Africa?: A scoping review | Doesn’t focus on reviewing interventions |
| Singh et al. 2018  *PLOS One* | Evaluating the effectiveness of sexual and reproductive health services during humanitarian crises: A systematic review | Doesn’t focus specifically on men and boys/male engagement |
| Suandi et al. 2020  *International Health* | Does involving male partners in antenatal care improve healthcare utilisation? Systematic review and meta-analysis of the published literature from low- and middle-income countries | Doesn’t focus on reviewing interventions |
| Tanner-Smith et al. 2015  *Journal of Youth and Adolescence* | Can brief alcohol interventions for youth also address concurrent illicit drug use? results from a meta-analysis | Doesn’t focus on SRHR per WHO framework |
| Tsegaye et al. 2020  *BMC Public Health* | The magnitude of adherence to Option B plus program and associated factors among women in eastern African countries: A systematic review and meta-analysis | Doesn’t focus specifically on men and boys/male engagement |
| Tucker et al. 2017  *EBioMedicine* | Enhancing public health HIV interventions: a qualitative meta-synthesis and systematic review of studies to improve linkage to care, adherence, and retention | Doesn’t focus specifically on men and boys/male engagement |
| Verbeek et al. 2023  *Archives of Sexual Behavior* | sexual and dating violence prevention programs for male youth: a systematic review of program characteristics, intended psychosexual outcomes, and effectiveness | Outside of geographic focus^a^ |
| Yargawa et al. 2015 *Journal of Epidemiology And Community Health* | Male involvement and maternal health outcomes: Systematic review and meta-analysis | Doesn’t focus on reviewing interventions |
| Zhang et al. 2021  *AIDS and Behavior* | Efficacy of psychological interventions towards the reduction of high-risk sexual behaviors among people living with HIV: A systematic review and meta-analysis, 2010–2020 | Outside of geographic focus^a^ |
| Zhang et al. 2023  *Journal of American College Health* | University-based behavioral interventions to promote safer sex practices: A systematic review and meta-analysis. | Outside of geographic focus^a^ |
| Zielke et al. 2023  *International Journal of Equity in Health* | Operationalising masculinities in theories and practices of gender-transformative health interventions: a scoping review | Outside of geographic focus^a^ |
| **Articles excluded at data extraction and quality review stage** | | |
| Aguiar et al. 2015  *Maternal Child Health J* | Impact of male partner antenatal accompaniment on perinatal health outcomes in developing countries: A systematic literature review | Inadequate quality of systematic review, per AMSTAR 2 criteria |
| Angusubalakshmi et al. 2023  *Indian Journal of Public Health* | male involvement as a significant contributor for enhancing maternal and child health-care services: a scoping review | Inadequate quality of systematic review, per AMSTAR 2 criteria |
| Casey et al. 2018  *Trauma, Violence, & Abuse* | Gender transformative approaches to engaging men in gender-based violence prevention: A review and conceptual model | Inadequate quality of systematic review, per AMSTAR 2 criteria |
| Emalia et al. 2023  *Jurnal Keperawatan Padjadjaran* | Couple-based interventions for secondary and tertiary prevention of intimate partner violence: A systematic review of randomized controlled trials | Inadequate quality of systematic review, per AMSTAR 2 criteria |
| Taliep et al. 2021  *Journal of Interpersonal Violence* | A qualitative meta-synthesis of interpersonal violence prevention programs focused on males | Inadequate quality of systematic review, per AMSTAR 2 criteria |

^a^ Did not include at least half of the primary studies from LMIC per World Bank definition (or conduct separate analysis of evidence from LMIC countries).

^b^ To be considered systematic the review had to meet all the following criteria: (1) The a priori specification of a research question; (2) clarity on the scope of the review and which studies are eligible for inclusion; (3) making every effort to find all relevant research and to ensure that issues of bias in included studies are accounted for; and (4) analyzing the included studies in order to draw conclusions based on all the identified research in an impartial and objective way.
