# Supplementary material for: Systematic review of reviews on interventions to engage men and boys as clients, partners and agents of change for improved sexual and reproductive health and rights
Source: BMJ Open. 2025 Jan 20;15(1):e083950. doi: 10.1136/bmjopen-2024-083950 (PMC11751930; doi:10.1136/bmjopen-2024-083950)
Supplement: Supplementary file 3 [file bmjopen-2024-083950.reviewer_comments.pdf]

PEER REVIEW HISTORY

BMJ Open publishes all reviews undertaken for accepted manuscripts. Reviewers are asked to complete a checklist review form and are provided with free text boxes to elaborate on their assessment. These free text comments are reproduced below.

ARTICLE DETAILS

Title (Provisional)

Systematic review of reviews on interventions to engage men and boys as clients, partners, and agents of change for improved sexual and reproductive health and rights

Authors

Gottert, Ann; Pulerwitz, Julie; Weiner, Renay; Okondo, Chantalle; Werner, Jesse; Magni, Sarah; Mathur, Sanyukta

---

VERSION 1 - REVIEW

**Reviewer**	**1**
**Name**	**Baroudi, Mazen**
**Affiliation**	**Umeå Universitet Medicinska fakulteten, Epidemiology and Global Health**
**Date**	**08-Apr-2024**
**COI**	**None to declare**

Thank you for inviting me to review this important paper. The paper tries to create a comprehensive summary of interventions targeting men as clients, partners and agents of change in the area of sexual and reproductive health and rights.

The main weakness of this review is validity and reproducibility.

• The review is neither pre-registered nor a protocol is published in any database.

• The review does not indicate how many articles are identified from each of the searched databases (PubMed, Cochrane, CINHAL, WoS).

• The authors need to provide search terms that other researchers can use to check the validity of their searches. The search strategy provided in “Supplemental file1” is flawed:

o Searching the all the blocks together in PubMed yield 0 records

o Searching the first block (systematic review block) in PubMed yields 604 records. Searching for only “systematic review”[ti] in PubMed yield 243,795 records. This is because the incorrectly added “AND” to this block which restricts the search rather than expanding it.

• It is not clear if the authors have updated their search in mid-2023. The author states that "The search was conducted between 18th October and 9th November 2022" but they also state that "The review also had to be published from 2015 to present (mid-2023)"

• It is not clear how the analysis of the extracted outputs went through. While the authors claim that "to identify emerging themes, then met together and then with the full study team to reach consensus on themes", they don't present any themes in their results except for the predefined sections of the framework (clients, partners, agents of change)

• Most of the results are presented without supporting references. One example of that is their statement "A few reviews concluded there was an insufficient volume of evidence in certain areas – particularly for reviews of men as family planning users, and for HIV self-testing (a more recent innovation)". To allow for the reader to fact-check the statement, each and every statement in the results should be followed by the supporting references.

• Although very informative, it is not clear how the authors developed figure 2. Many of the statement in figure 2 are not presented at all in the previous sections and therefor have no supporting refences.

Another issue is the focus of the focus of the review which needs more clarification/justification. The authors state that on the review "include a focus on interventions that explicitly sought to engage heterosexual men and/or boys; and cover at least one of seven SRHR domains. WHO domains" "SRHR domains (Table 2) were defined following the WHO Reproductive Health Strategy.[7]". Reference 7: (Reproductive health strategy to accelerate progress towards the attainment of international development goals and targets) does not provide any SRHR domains rather identifies 5 priorities including infertility (which is not included in the review). The authors should provide a correct reference and a clear justification of how they identify these 7 domains. The current focus leaves behind very important SRHR topics that concern men as clients such as sexual dysfunction, prostatic and testicular cancers, and infertility.

**Reviewer**	**2**
**Name**	**Grubb, Laura**
**Affiliation**	**Boston Children's Hospital, Adolescent Medicine**
**Date**	**08-Apr-2024**
**COI**	**none**

Appreciate the opportunity to review this ambitious and much-needed study/review. The strengths of this manuscript include large number of studies, careful analysis and grouping of themes/outcomes, and effort to fill in much needed gaps in the literature. Ultimately, with

significant revision, this will be an excellent manuscript for publication. Overall, it is well written.

A couple of areas for improvement include:

• Reduce use of passive verb tense and increase use of active verb tense to provide more concise and stronger writing (https://www.grammarly.com/blog/active-vs-passive-voice/)

• Provide a definition for sexual and reproductive health and rights

• Provide an age range for boys and men

• Clarify early on in the manuscript that the subjects are cisgender (?) males who participate in sex with cisgender females

• Avoid equating heterosexual activity (sex between males and females) to heterosexual identity. Just use the behavior you are studying.

• Provide definition for family planning and consider if this is the best term (it might be)- in some cultures this term may have negative connotations. Consider other terms such as child spacing, birth spacing, contraception, etc.
(https://www.thelancet.com/journals/langlo/article/PIIS2214-109X(13)70177-3/fulltext)

• Please provide the SRHR domains in the Introduction or Methods and the origin of this defined domains

• Please provide inclusion and exclusion criteria in the Methods since you mention inclusion/exclusion in results- some considerations include: language, ages, geography, specific countries/continents, etc.

• On page 3, line 7-8, authors say, "It is hoped that this will facilitate scale-up and integration of these strategies for improved public health impact." What do you mean by improved public health impact? What are some positive outcomes?

• What were the qualifications of this systematic review's reviewers?

• There is a heavy emphasis on HIV and circumcision.. please provide back background information/context for this.

• What about other important male SRH metrics such as males as victims of violence? Other STIs besides HIV?

• Define middle to low income countries

• Use substance use instead of drug abuse (less stigmatizing)- page 11, line 43

• Page 12, line 12 authors write about men at highest risk- please provide definition or characteristics which categorize as highest risk

• Paper is text heavy, recommend summarizing more of findings in tables, graphs, or other graphics

**Reviewer**	**3**
**Name**	**Thompson Coon, Jo**
**Affiliation**	**University of Exeter Medical School, NIHR CLAHRC South West Peninsula**
**Date**	**30-Jul-2024**
**COI**	**None.**

This is a well conducted overview of systematic reviews which aims to improve understanding of interventions to engage men and boys to improve sexual and reproductive health and rights. The overview includes 31 systematic review and uses an existing framework to organise the findings into three categories - programmes for men and boys as clients, as partners and as agents of change. The authors conclude that there is substantial evidence to support a range of successful interventions to engage men and boys to improve SRHR, with markedly similar principles and approaches across domains.

Major comments:

1. In the background, the authors suggest that there may be adverse effects of these interventions e.g. harming women's sexual reproductive rights and health. In the body of the paper, the authors report that there was no evidence of adverse impacts of the interventions - the abstract would be strengthened by the inclusion of this finding.

2. Relatedly, as this is a review of reviews, the authors are reliant on the aims/objectives of the included reviews aligning with the aims and objectives of the overview. It isn't clear whether consideration of adverse events was a focus of all included reviews. The authors should reflect on this within the discussion and also potentially within the limitations as an overview of reviews has the potential to further amplify any bias within included reviews.

3. The searches were conducted in November 2022 and are therefore nearly two years out of date. The authors state that they 'became aware' of a relevant review published in 2023 but not whether they conducted any further update searches or citation checking. It is considered best practice for searches for systematic reviews to be less than 12 months old at the date of publication. The authors should therefore update the search. Use of a search summary table (https://pubmed.ncbi.nlm.nih.gov/33424470/) may be helpful to expedite this process.

4. An indication of the degree of overlap between reviews e.g. how many unique primary studies are included and how many of the reviews include the same studies would be helpful for the reader since overlapping reviews/overreliance on a small number of primary studies represented in multiple reviews can be a source of bias.

5. Figure 2 is an interesting addition to the paper, however, it would be more meaningful if the methodological detail on how the figure was produced was included within the methods section.

6. The section on patient and public involvement is great to see. However, it isn't clear from the detail provided who was involved and how. Who were the stakeholders, what activities were they involved in and when? Were any patients involved? Further detail would strenghten this element of the paper. The authors might want to consider using the short form of the GRIPP2 checklist https://researchinvolvement.biomedcentral.com/articles/10.1186/s40900-017-0062-2/tables/2 to guide reporting of patient and public involvement.

7. The authors should ensure that the paper complies with PRISMA reporting guidance and provide a completed checklist.

Minor comments:

1. In the search description, the authors should clarify what they searched in 'Cochrane' - was this the Cochrane Library? Or the CDSR? Or something else?

2. I note that the authors did not conduct any supplementary searching e.g. web searching, or citation chasing of included reviews. This should be considered a limitation of the method and included within the discussion of limitations.

3. Please consider whether Mary Chu who provided support with database searches has (or could) contributed sufficiently to warrant authorship since the searches are fundamental to the success of any evidence synthesis.

VERSION 1 - AUTHOR RESPONSE

Reviewer: 1
Dr. Mazen Baroudi, Umeå Universitet Medicinska fakulteten
Comments to the Author:
Thank you for inviting me to review this important paper. The paper tries to create a comprehensive summary of interventions targeting men as clients, partners and agents of change in the area of sexual and reproductive health and rights.

The main weakness of this review is validity and reproducibility.

• The review is neither pre-registered nor a protocol is published in any database.
RESPONSE: We had developed a detailed protocol for the review, which was referenced internally. We did not prospectively register the review, but have now registered the review on Research Registry (unique identification number (UIN): reviewregistry1884). This registration is now stated in the first paragraph of the Methods section.

• The review does not indicate how many articles are identified from each of the searched databases (PubMed, Cochrane, CINHAL, WoS).
RESPONSE: We have now included the number of articles identified form each

database (at both timepoints of our search- late 2022 and September 2024) in Supplemental file 1.

• The authors need to provide search terms that other researchers can use to check the validity of their searches. The search strategy provided in "Supplemental file1" is flawed:
o Searching the all the blocks together in PubMed yield 0 records
o Searching the first block (systematic review block) in PubMed yields 604 records. Searching for only "systematic review"[ti] in PubMed yield 243,795 records. This is because the incorrectly added "AND" to this block which restricts the search rather than expanding it.
RESPONSE: We have now included and updated this information in Supplemental file 1.

• It is not clear if the authors have updated their search in mid-2023. The author states that "The search was conducted between 18th October and 9th November 2022" but they also state that "The review also had to be published from 2015 to present (mid-2023)"
RESPONSE: As noted above, we have updated the search to add reviews published between 9th November 2022 (the day after the end date of our last search) to 31 December 2023. This resulted in 4 new reviews being added, for a total of 35 reviews. We have integrated descriptions of the procedures for this updated review, and updated findings and discussion, in the manuscript. Of note, the 4 new reviews largely reflected similar findings and conclusions, as the previous 31.

• It is not clear how the analysis of the extracted outputs went through. While the authors claim that "to identify emerging themes, then met together and then with the full study team to reach consensus on themes", they don't present any themes in their results except for the predefined
sections of the framework (clients, partners, agents of change)
RESPONSE: Thanks for this suggestion. We have now added more detail to the description of how analysis of the extracted outputs proceeded.

• Most of the results are presented without supporting references. One example of that is their statement "A few reviews concluded there was an insufficient volume of evidence in certain areas – particularly for reviews of men as family planning users, and for HIV self-testing (a more recent innovation)". To allow for the reader to fact-check the statement, each and every statement in the results should be followed by the supporting references.
RESPONSE: We have added in several additional references to the many that are already included throughout the results section (including for the particular instance noted). In some cases, for broader statements that apply to most reviews, we opted not to include all possible citations for parsimony and to improve readability.

• Although very informative, it is not clear how the authors developed figure 2. Many of the statement in figure 2 are not presented at all in the previous sections and therefor have no supporting refences.
RESPONSE: We agree with the need to better explain how and why we developed the conceptual framework in Figure 2. We have now added a description of this in the methods section at the end of the "Data extraction and analysis" sub-section. It reads: "Finally, we sought to develop a summative conceptual framework that identifies the types of male engagement interventions and strategies most consistently found to be effective across the reviews, mapping onto the men as clients/partners/agents of change categories as well as different levels of the social ecological framework. This conceptual framework is intended to be a future reference for programmers, researchers, policymakers, and other stakeholders who share the goal of improving SRHR for all." In addition, we have further refined Figure 2 to most directly reflect review findings, and have modified its format to appear more like a table which we

believe will make it more straightforward for readers to link findings described in the results section, to the framework.

Another issue is the focus of the focus of the review which needs more clarification/justification. The authors state that on the review “include a focus on interventions that explicitly sought to engage heterosexual men and/or boys; and cover at least one of seven SRHR domains. WHO domains” “SRHR domains (Table 2) were defined following the WHO Reproductive Health Strategy.[7]”. Reference 7: (Reproductive health strategy to accelerate progress towards the attainment of international development goals and targets) does not provide any SRHR domains rather identifies 5 priorities including infertility (which is not included in the review). The authors should provide a correct reference and a clear justification of how they identify these 7 domains. The current focus leaves behind very important SRHR topics that concern men as clients such as sexual dysfunction, prostatic and testicular cancers, and infertility.
RESPONSE: We have now described the source of the 7 domains more accurately. Previously, we had stated that “SRHR domains (Table 2) were defined following the WHO Reproductive Health Strategy” (and cited the 2004 WHO Reproductive Health Strategy). We have revised to read: “SRHR domains (Table 2) were defined based on outcomes of interest per the WHO Reproductive Health Strategy, as employed by Ruane-McAteer et al. (2019) in their WHO-commissioned evidence and gap map of interventions engaging men and boys in SRHR.” We now cite both the 2004 WHO Reproductive Health Strategy document as well as Ruane-McAteer et al. (2019). We have also included more detail about each SRHR domain in Table 2, which helps clarify that outcomes like infertility and sexual dysfunction (as raised by the reviewer) are in fact included. Note that prostatic and testicular cancers are not included since they are not acquired sexually, per Ruane-McAteer et al. (this is now noted under Table 2).

Reviewer: 2
Dr. Laura Grubb, Boston Children's Hospital
Comments to the Author:
Appreciate the opportunity to review this ambitious and much-needed study/review. The strengths of this manuscript include large number of studies, careful analysis and grouping of themes/outcomes, and effort to fill in much needed gaps in the literature. Ultimately, with significant revision, this will be an excellent manuscript for publication. Overall, it is well written.

A couple of areas for improvement include:

• Reduce use of passive verb tense and increase use of active verb tense to provide more concise and stronger writing (https://www.grammarly.com/blog/active-vs-passive-voice/)
RESPONSE: We have tried to change from passive to active tense whenever possible throughout the paper.

• Provide a definition for sexual and reproductive health and rights
RESPONSE: Thank you for suggesting this. We have added a definition at the start of the second paragraph of the introduction (using the definition proposed by the Guttmacher–Lancet Commission (Starrs et al. 2018)).

• Provide an age range for boys and men
RESPONSE: Thank you for this suggestion; this was an oversight. We have now specified the age range of 10 years or older, in the last paragraph of the introduction, and in the “Search and eligibility criteria” subsection of the methods section.

• Clarify early on in the manuscript that the subjects are cisgender (?) males who participate in sex with cisgender females
RESPONSE: We have further refined the sentence we had included on this topic in the last paragraph of the introduction section, to reflect the approach we took for the review. The included reviews often did not specify a focus on cisgender males (or who participate in sex with cisgender females). Formerly, this sentence read: "We focused our analysis on interventions to engage heterosexual men to improve SRHR, since there is another sizable literature on sexual and gender minorities' engagement around SRHR, and the types of programming, and effectiveness, may differ between these populations." It now reads: "We did not include reviews focused on sexual and/or gender minority men's engagement in SRHR, since there is another sizable literature on this topic, and the types of programming, and effectiveness, may differ for these populations."

• Avoid equating heterosexual activity (sex between males and females) to heterosexual identity. Just use the behavior you are studying.
RESPONSE: We have removed the few instances where we emphasize heterosexual, as in fact often reviews did not specify per se a focus on heterosexual men. Instead, we have added the following statement (complementing the sentence already at the end of the introduction – see response above), in the methods section: "An exclusion criterion was if the review primarily focused on sexual and/or gender minority populations, for the reasons described above."

• Provide definition for family planning and consider if this is the best term (it might be)- in some cultures this term may have negative connotations. Consider other terms such as child spacing, birth spacing, contraception, etc.
(https://www.thelancet.com/journals/langlo/article/PIIS2214-109X(13)70177-3/fulltext)
RESPONSE: We sought to reflect the terms used in the included reviews, whenever possible. Various terms related to family planning were employed in the reviews, particularly contraception. Hence, we have added that term alongside family planning in several instances throughout the paper.

• Please provide the SRHR domains in the Introduction or Methods and the origin of this defined domains
RESPONSE: We have further clarified the origin of the defined domains in the methods section (under the subsection "Search and eligibility criteria"). We have added the list of domains in the text in this section as well and have expanded descriptions of the domains in Table 2.

• Please provide inclusion and exclusion criteria in the Methods since you mention inclusion/exclusion in results- some considerations include: language, ages, geography, specific countries/continents, etc.
RESPONSE: We believe we have already included this information, under the subsection "Search and eligibility criteria".

• On page 3, line 7-8, authors say, "It is hoped that this will facilitate scale-up and integration of these strategies for improved public health impact." What do you mean by improved public health impact? What are some positive outcomes?
RESPONSE: This particular sentence was from the "Key questions" summary section which we have now removed as it is not required by BMJ Open. However, in similar wording elsewhere we have now specified that we mean public health impact on SRHR outcomes.

• What were the qualifications of this systematic review's reviewers?

RESPONSE: We have added the following text in the methods section: "All reviewers have experience with SRHR programming and research, as well as synthesis of SRHR evidence, in sub-Saharan Africa, and globally (for several reviewers). AG & JP each have 20+ years of experience developing, implementing, and evaluating programs to engage men and boys in SRHR worldwide, have developed frameworks (including the men as clients/partners/agents of change framework employed in this review), and have published extensively regarding male engagement in SRHR." We also included several references to support the last statement.

• There is a heavy emphasis on HIV and circumcision.. please provide back background information/context for this.
RESPONSE: As this is a systematic review of reviews, the emphasis depends upon what reviews were identified via the search – which focused quite heavily on HIV. We now note this as a limitation, and also identify the need for further research in other SRHR areas.

• What about other important male SRH metrics such as males as victims of violence? Other STIs besides HIV?
RESPONSE: Thank you. We have further described (in the discussion section) SRHR areas/outcomes in need of further research/summarizing via systematic reviews, including men as victims of sexual and gender-based violence, and other STIs besides HIV.

• Define middle to low income countries
RESPONSE: We had specified that LMIC were defined based on World Bank criteria; those criteria are complex and describing them would take a lot of text, however we have added a citation to a World Bank webpage where this description is offered.

• Use substance use instead of drug abuse (less stigmatizing)- page 11, line 43
RESPONSE: Thank you; we have made this change.

• Page 12, line 12 authors write about men at highest risk- please provide definition or characteristics which categorize as highest risk
RESPONSE: We have now tried to clarify this statement by adding the underlined text in the sentence: "Moreover, while interventions were successful overall, it is often not clear how true this was for men at highest risk <u>of poor SRHR outcomes for themselves and/or their partners/families, who are often not easily identifiable</u> and may also be hardest to reach." We have added a few more references to support this statement as well.

• Paper is text heavy, recommend summarizing more of findings in tables, graphs, or other graphics
RESPONSE: We appreciate the reviewer's concern. The nature of a systematic review of reviews requires quite a bit of textual explanation. We have included Figure 2 as a way of summarizing results. We have also tried to condense wording in text whenever possible.

Reviewer: 3
Dr. Jo Thompson Coon, University of Exeter Medical School
Comments to the Author:
This is a well conducted overview of systematic reviews which aims to improve understanding of interventions to engage men and boys to improve sexual and reproductive health and rights. The overview includes 31 systematic review and uses an existing framework to organise the findings into three categories - programmes for men and boys as

clients, as partners and as agents of change. The authors conclude that there is substantial evidence to support a range of successful interventions to engage men and boys to improve SRHR, with markedly similar principles and approaches across domains.

Major comments:

1. In the background, the authors suggest that there may be adverse effects of these interventions e.g. harming women's sexual reproductive rights and health. In the body of the paper, the authors report that there was no evidence of adverse impacts of the interventions - the abstract would be strengthened by the inclusion of this finding.
RESPONSE: We agree this is an important point to mention in the abstract. The abstract already states that: "no adverse intervention impacts on prevalence of SRHR outcomes were reported" (noting we changed to the word "reported" from "found").

2. Relatedly, as this is a review of reviews, the authors are reliant on the aims/objectives of the included reviews aligning with the aims and objectives of the overview. It isn't clear whether consideration of adverse events was a focus of all included reviews. The authors should reflect on this within the discussion and also potentially within the limitations as an overview of reviews has the potential to further amplify any bias within included reviews.
RESPONSE: We have added a statement in the limitations section reminding readers of the reliance of the review of reviews on the included reviews. "…findings from a systematic review of reviews are necessarily reflective of the set of systematic reviews available for inclusion. For instance, there was a heavy focus on HIV services in our review, given so many included reviews focused on this topic. The magnitude and breadth of included reviews helps assuage this concern." We have also added the following underlined phrasing to a sentence in the second paragraph of the discussion section where we describe review findings re: adverse impacts of interventions: "Most, although not all, reviews appeared to have assessed adverse effects reported in primary studies, <u>although this was rarely a primary objective</u>."

3. The searches were conducted in November 2022 and are therefore nearly two years out of date. The authors state that they 'became aware' of a relevant review published in 2023 but not whether they conducted any further update searches or citation checking. It is considered best practice for searches for systematic reviews to be less than 12 months old at the date of publication. The authors should therefore update the search. Use of a search summary table (https://pubmed.ncbi.nlm.nih.gov/33424470/) may be helpful to expedite this process.
RESPONSE: We have updated the search to add reviews published between 9th November 2022 (the day after the end date of our last search) to 31 December 2023. This resulted in 4 new reviews being added, for a total of 35 reviews. We have integrated descriptions of the procedures for this updated review, and updated findings and discussion, in the manuscript. Of note, the 4 new reviews largely reflected similar findings and conclusions, as the previous 31.

4. An indication of the degree of overlap between reviews e.g. how many unique primary studies are included and how many of the reviews include the same studies would be helpful for the reader since overlapping reviews/overreliance on a small number of primary studies represented in multiple reviews can be a source of bias.
RESPONSE: Thank you for this suggestion. We have now clarified, in the results section, the degree of overlap in primary studies by (1) stating the total number of *non-overlapping* primary studies (under the section "Characteristics of included reviews") – namely, 966 of the 1,080 total primary papers were non-overlapping, representing a relatively small degree of overlap; (2) Under the section "Quality and risk of bias of the reviews", identifying several primary studies that were over-represented, which tended to fall within the "Men as agents of change" category.

5. Figure 2 is an interesting addition to the paper, however, it would be more meaningful if the methodological detail on how the figure was produced was included within the methods section.
RESPONSE: As stated above in relation to a comment from Reviewer 1, we agree with the need to better explain how and why we developed the conceptual framework in Figure 2. We have now added a description of this in the methods section at the end of the "Data extraction and analysis" sub-section. It reads: "Finally, we sought to develop a summative conceptual framework that identifies the types of male engagement interventions and strategies most consistently found to be effective across the reviews, mapping onto the men as clients/partners/agents of change categories as well as different levels of the social ecological framework. This conceptual framework is intended to be a future reference for programmers, researchers, policymakers, and other stakeholders who share the goal of improving SRHR for all." In addition, we have further refined Figure 2 to most directly reflect review findings and have modified its format to appear more like a table which we believe will make it more straightforward for readers to link findings described in the results section, to the framework.

6. The section on patient and public involvement is great to see. However, it isn't clear from the detail provided who was involved and how. Who were the stakeholders, what activities were they involved in and when? Were any patients involved? Further detail would strenghten this element of the paper. The authors might want to consider using the short form of the GRIPP2 checklist https://researchinvolvement.biomedcentral.com/articles/10.1186/s40900-017-0062-2/tables/2 to guide reporting of patient and public involvement.
RESPONSE: Thanks for this suggestion. We have added more details to our description of who was involved and how.

7. The authors should ensure that the paper complies with PRISMA reporting guidance and provide a completed checklist.
RESPONSE: We have now included a completed PRIMSA checklist in our submission.

Minor comments:

1. In the search description, the authors should clarify what they searched in 'Cochrane' - was this the Cochrane Library? Or the CDSR? Or something else?
RESPONSE: Thank you for this request. We have now clarified in the manuscript and Supplemental file 1, that we searched the Cochrane Database of Systematic Reviews.

2. I note that the authors did not conduct any supplementary searching e.g. web searching, or citation chasing of included reviews. This should be considered a limitation of the method and included within the discussion of limitations.
RESPONSE: Thank you for pointing this out. We did in fact conduct supplementary searching and omitted describing this in error. We have now added the following statement to the methods section under Search and eligibility criteria: "We conducted supplementary searching to identify any further reviews which had not been found in the database searches, including hand-searches of reference lists of included articles."

3. Please consider whether Mary Chu who provided support with database searches has (or could) contributed sufficiently to warrant authorship since the searches are fundamental to

the success of any evidence synthesis.
RESPONSE: We appreciate this suggestion and have considered whether to add Mary Chu as a co-author, including in relation to ICJME authorship guidelines. She had a more limited role in this particular review, limited to conducting the database search, as compared to the role librarians sometimes play in systematic reviews (in which case the librarian may be involved in conceptualizing the research questions, designing the search strategy, conducting abstract/full text reviews, data extraction, etc). Therefore we decided not to include her as co-author, but continue to acknowledge her contributions in the acknowledgments section.

VERSION 2 - REVIEW

**Reviewer**	**2**
**Name**	**Grubb, Laura**
**Affiliation**	**Boston Children's Hospital, Adolescent Medicine**
**Date**	**06-Nov-2024**
**COI**	

Thank you to the authors for these excellent revisions. This reviewer provides a few additional recommendations for revision.

Page 2, line 54 consider changing to say, "We aimed to summarize evidence…"

Page 4, line 7 please define AG and JP as this is the first reference to them

Table 2 lists disease outbreak such as zika or ebola. Was there anything regarding MPox?

Table 2 lists as below. Will you please provide ages or definitions for adults versus youth versus men versus young men? There may be variability in these terms in different regions or cultures, so wondering who exactly the authors are referring to.

Main focal population(s) for interventions

Both adults and youth 17 49%

Adults 11 31%

Youth 3 9%

Men and young men

Page 11, line 32 uses the term "adolescent boys and young men" please define the ages in this group.

Page 12, line 5, consider using external condoms instead of male condoms as this is a more inclusive term for different types of sexual activity.

By page 12, I found it too hard to keep track of all the abbreviations. Perhaps continue to use the long form for less common or recognizable terms.

The text portion of the Results is still quite long and cumbersome to read through. Are you able to summarize more succinctly, perhaps with graphics?

Reviewer **3**

Name **Thompson Coon, Jo**

Affiliation **University of Exeter Medical School, NIHR CLAHRC South West Peninsula**

Date **11-Nov-2024**

COI

Thank you for your comprehensive response to reviewer comments. I have no further comments.

VERSION 2 - AUTHOR RESPONSE

Reviewer: 2
Dr. Laura Grubb, Boston Children's Hospital
Comments to the Author:
Thank you to the authors for these excellent revisions. This reviewer provides a few additional recommendations for revision.

Page 2, line 54 consider changing to say, "We aimed to summarize evidence…"

RESPONSE: This sentence is meant to describe what a systematic review of reviews is, but agree this is not clear given the sentence's current placement. We have moved this sentence up to follow the first sentence in the paragraph in which we state that we conducted a systematic review of reviews.

Page 4, line 7 please define AG and JP as this is the first reference to them
RESPONSE: Thanks for this suggestion. We now have expanded this to read "Authors A.G. and J.P. each…"

Table 2 lists disease outbreak such as zika or ebola. Was there anything regarding MPox?
RESPONSE: Thank you; the previous wording wrongly suggested that Zika and Ebola are the only relevant viruses. We have changed the row entry from "Promoting SRHR in disease outbreaks (including prevention of sexual transmission of Zika and Ebola viruses)" to read "Promoting SRHR in disease outbreaks (including prevention of sexual transmission, e.g., of Zika or Ebola viruses)".

Table 2 lists as below. Will you please provide ages or definitions for adults versus youth versus men versus young men? There may be variability in these terms in different regions or cultures, so wondering who exactly the authors are referring to.

Main focal population(s) for interventions
Both adults and youth 17 49%
Adults 11 31%
Youth 3 9%
Men and young men
RESPONSE: Thanks for this helpful request for clarification. We have added the following footnote :"By "youth"/"young men" is meant ages 10-24"

Page 11, line 32 uses the term "adolescent boys and young men" please define the ages in this group.

RESPONSE: Thanks for this request; when we went back to the reviews concerning VMMC, we saw that most often adolescent boys, vs. adolescent boys and young men, was most often used (e.g., in comparison with adult men). We have therefore taken out "young men" and clarified that most often, adolescent men were described as being ages 10-19 years.
Page 12, line 5, consider using external condoms instead of male condoms as this is a more inclusive term for different types of sexual activity.

RESPONSE: We have revised "male condoms" to "male/external condoms"
By page 12, I found it too hard to keep track of all the abbreviations. Perhaps continue to use the long form for less common or recognizable terms.
RESPONSE: We have now removed several abbreviations throughout the manuscript, particularly those that are less common/recognizable or those used only a few times.

The text portion of the Results is still quite long and cumbersome to read through. Are you able to summarize more succinctly, perhaps with graphics?
RESPONSE: We have now added several sub-headings within the three main sections (Men as clients/partners/agents of change), which we hope will help provide more structure and help break up these sections.

Reviewer: 3
Dr. Jo Thompson Coon, University of Exeter Medical School
Comments to the Author:
Thank you for your comprehensive response to reviewer comments. I have no further comments.

****Please note that in addition to the revisions described above, we have taken the liberty of slightly revising certain text in Figure 2 (the conceptual framework) to best align with review findings – and thus have reattached Figure 2 to this submission.**
